# Boosting Oxygen Evolution Reaction Performance on NiFe-Based Catalysts Through *d*-Orbital Hybridization

**DOI:** 10.1007/s40820-024-01528-9

**Published:** 2024-09-26

**Authors:** Xing Wang, Wei Pi, Sheng Hu, Haifeng Bao, Na Yao, Wei Luo

**Affiliations:** 1 State Key Laboratory of New Textile Materials and Advanced Processing Technology, Key Laboratory of New Textile Materials and Applications of Hubei Province, School of Materials Science and Engineering, Wuhan Textile University, Wuhan, 430200 People’s Republic of China; 2 College of Chemistry and Molecular Sciences, Wuhan University, Wuhan, 430072 People’s Republic of China

**Keywords:** NiFe-based catalysts, *d*-orbital coupling, Oxygen evolution reaction, Anion exchange membrane electrolyzer

## Abstract

Anion-exchange membrane water electrolyzers (AEMWEs) for green hydrogen production have received intensive attention due to their feasibility of using earth-abundant NiFe-based catalysts. By introducing a third metal into NiFe-based catalysts to construct asymmetrical M-NiFe units, the *d*-orbital and electronic structures can be adjusted, which is an important strategy to achieve sufficient oxygen evolution reaction (OER) performance in AEMWEs. Herein, the ternary NiFeM (M: La, Mo) catalysts featured with distinct M-NiFe units and varying *d*-orbitals are reported in this work. Experimental and theoretical calculation results reveal that the doping of La leads to optimized hybridization between *d* orbital in NiFeM and 2*p* in oxygen, resulting in enhanced adsorption strength of oxygen intermediates, and reduced rate-determining step energy barrier, which is responsible for the enhanced OER performance. More critically, the obtained NiFeLa catalyst only requires 1.58 V to reach 1 A cm^−2^ in an anion exchange membrane electrolyzer and demonstrates excellent long-term stability of up to 600 h.

**Supplementary Information:**

The online version contains supplementary material available at 10.1007/s40820-024-01528-9.

## Highlights

- The NiFeLa catalyst with 3*d*-5*d* orbital coupling exhibits remarkable oxygen evolution reaction (OER) activity and stability, enabling an anion-exchange membrane water electrolyzers device to achieve a cell voltage of only 1.58 V at 1 A cm^−2^ as well as long-term stability over 600 h.
- The introduction of La disrupts the symmetry of Ni-Fe units and optimize d band center, which affects the *d*-*p* orbital hybridization between the metal sites on the surface of the catalyst and oxygen-containing intermediates during the OER process.
- The 5*d*-introduced NiFeLa has enhanced adsorption strength of oxygen intermediates, which can reduce the rate-determining step energy barrier and prevent catalyst dissolution.

**Supplementary Information:**

The online version contains supplementary material available at 10.1007/s40820-024-01528-9.

## Graphical abstract

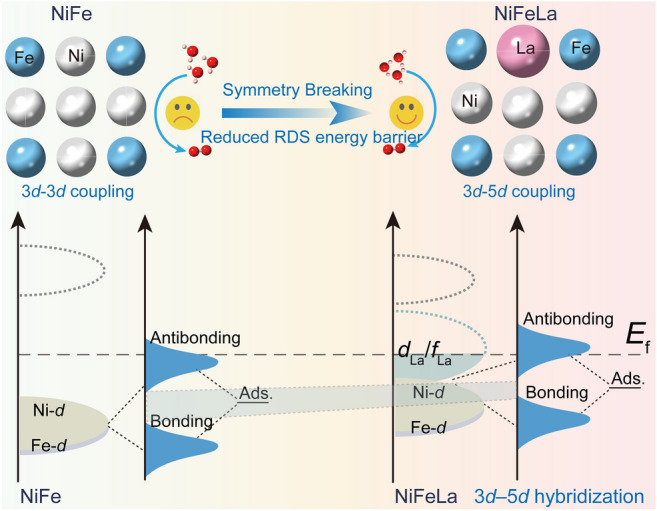

## Introduction

Oxygen evolution reaction (OER), a pivotal role in exploiting clean and renewable hydrogen, offers a pathway to reduce the dependence on fossil fuels and achieve carbon neutrality [1–5]. Essentially, the OER process accompanies four proton-coupled electrochemical reactions, typically resulting in sluggish reaction kinetics and poor electrochemical performance [6–8]. In the past decades, substantial scholarly efforts have been dedicated to the refinement of OER electrocatalysts, aimed at optimizing the intricate four-electron transfer reaction pathways, featuring materials such as transition metal oxides, layered structures, and perovskites [9–11]. In recent years, NiFe-based catalysts (e.g., (oxy) hydroxides, alloys, coordination polymers, nitrides, sulfides, and carbides) have attracted widespread attention [12–17]. However, their OER activities still fall short of expectations due to the higher energy barrier associated with the rate-determining step (RDS) [18–20]. Therefore, it is still urgent to discover effective approaches to overcome the sluggish RDS energy barrier, and further improve the OER performance of NiFe-based OER electrocatalysts.

Currently, the electronic modulation strategy provides rational direction in optimizing the intrinsic activity of electrocatalysts by balancing the adsorption of intermediates [12, 21, 22]. For NiFe-based catalysts, the adsorption of oxygen intermediates is primarily correlated with the *d*-band electron structure, which is mainly determined by the *d*-orbital structure [23–25]. Due to the symmetry breaking caused by the doping of different atoms, the *d* orbital structure can be rational regulated [26, 27]. For example, Fu and co-workers achieved high activity and stability of P-Ce SAS@CoO materials by constructing the asymmetric Co–O–Ce unit sites. They found that the enhanced adsorption of oxygen intermediates by optimizing the Co-3*d* orbital electron structure through *d-f* gradient orbital coupling was responsible for the enhanced OER activity [28]. Considering different positions of *5d*/*4d/3d*/*3d* energy levels relative to *E*_*f*_, as well as various orbital coupling effects, it is expected that different elements will construct different asymmetric M-M unit sites, and optimize *d*-orbital structures [29–32]. Despite considerable efforts have been achieved, the catalytic mechanism based on the different *5d*/*4d*/*3d-*introduced orbital and electronic structures for RDS during OER process have rarely been investigated.

In this work, we have successfully manufactured a new class of NiFeM (*M* = Mo, La) electrocatalysts via electrodeposition method. In particular, 5*d-*La introduced NiFeLa displays an ultralow overpotential of 190 mV at 10 mA cm^−2^ and an ultralong stability of 600 h at 100 mA cm^−2^ in 1 M KOH. Impressively, an anion-exchange membrane water electrolyzer (AEMWE) using NiFeLa as anode catalyst can operate stably for 600 h at 1 A cm^−2^. Experimental and theoretical calculations show that the introduction of La atoms into NiFe disrupts the symmetry of the Ni–Fe units and constructs 3*d*-5*d* orbital coupled Ni/Fe-M asymmetric geometric structure, leading to optimized *d*-*p* orbital hybridization between the metal sites on the surface of the catalyst and oxygen-containing intermediates, as well as promoted electron transfer and adsorption of oxygen intermediates.

## Experimental Section

### Materials

All chemicals were used as received. Ferric chloride hexahydrate (FeCl_3_·6H_2_O, ≥ 99%), Nickel chloride hexahydrate (NiCl_2_·6H_2_O, ≥ 97%), Sodium chloride (NaCl, ≥ 99.8%), Sodium molybdate anhydrous (Na_2_MoO_4_, ≥ 99%), Lanthanum nitrate hexahydrate (La(NO_3_)_3_·6H_2_O, ≥ 99%) were purchased from Sinopharm Chemical Reagent Co., Ltd. The carbon cloth (WOS 1011) was obtained from CeTech. Milli-Q purification system was used to obtain high-purity water.

### Fabrication of NiFeLa Alloys with Varying Amounts of La

15 mmol of NiCl_2_·6H_2_O, 5 mmol of FeCl_3_·6H_2_O, 10 mmol of NaCl and a certain amount of La(NO_3_)_3_·6H_2_O (0.25, 0.5, 1, and 1.5 mmol) were dissolved in 100 mL ultrapure water. A graphite rod was used as counter electrode, a standard Hg/HgO electrode was used as reference electrode, and carbon cloth was used as the working electrode. Carbon cloth was subjected to hydrophilic treatment before use, using a tube furnace annealing at 450 °C for 150 min. The samples were prepared by electrodeposition method at a current density of −100 mA cm^−2^ for 200 s. The samples synthesized by adding 0.25, 0.5, 1, and 1.5 mmol La(NO_3_)_3_·6H_2_O were named as NiFeLa0.25, NiFeLa, NiFeLa1 and NiFeLa1.5, respectively.

### Fabrication of NiFeMo Alloy

The synthesis of NiFeMo follows the same procedure as the above-mentioned synthesis method, with the deposition solution consisting of 0.5 mmol Na_2_MoO_4_.

### Fabrication of NiFe Alloy

The synthesis of NiFe alloy follows the same procedure as the above-mentioned synthesis method, with the deposition solution consisting of only 15 mmol of NiCl_2_·6H_2_O, 5 mmol of FeCl_3_·6H_2_O and 10 mmol of NaCl.

Details on material characterizations, electrochemical measurements and computation methods are provided in supporting information.

## Results and Discussion

### Characterizations of the Prepared Electrocatalysts

**Fig. 1:**
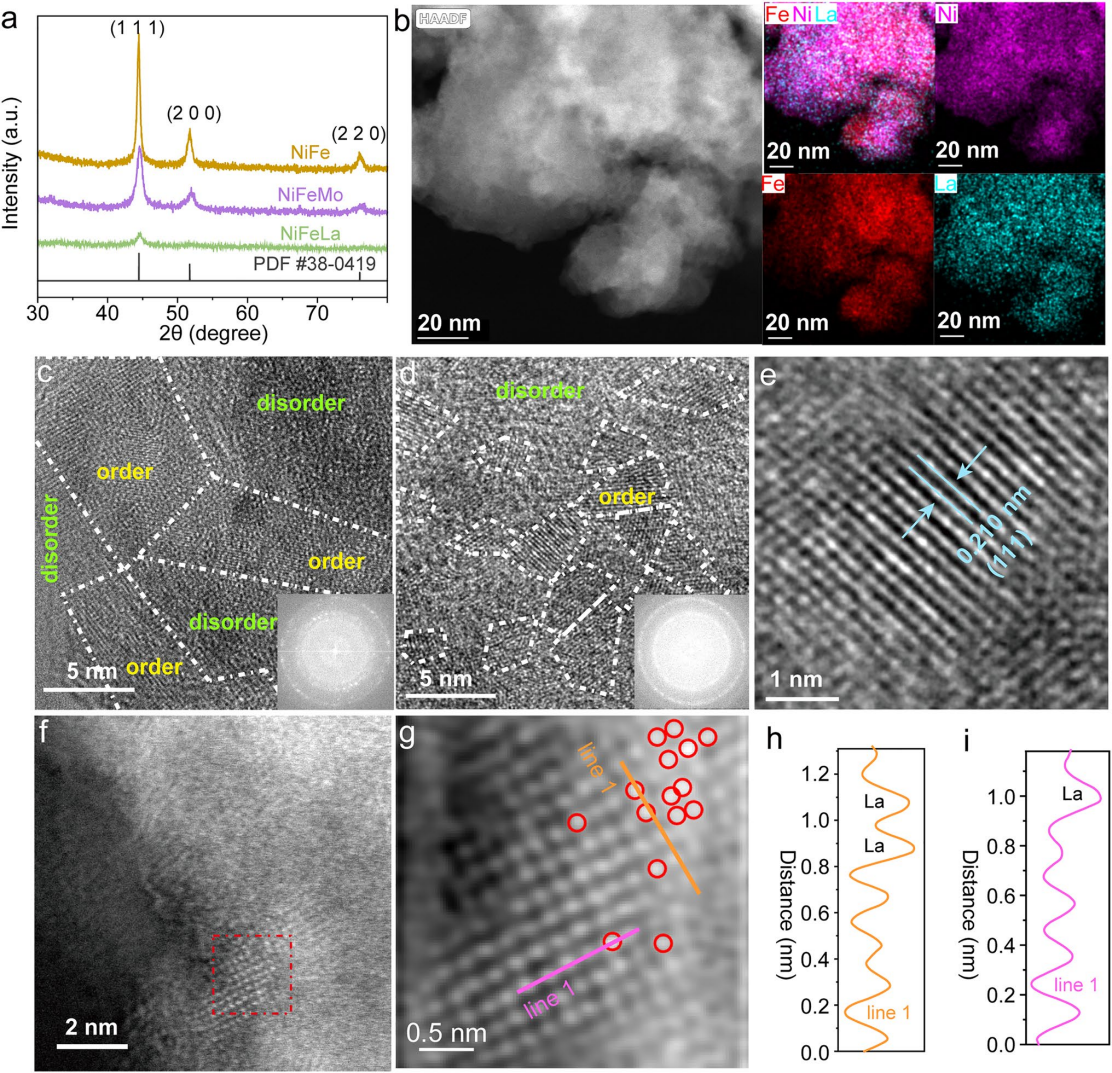
**a** The XRD patterns of NiFe, NiFeMo and NiFeLa. **b** HAADF-STEM image with elemental mappings of NiFeLa. **c** HRTEM image of NiFeMo. **d, e** HRTEM image of NiFeLa. The insets in (**c, d**) show the FFT patterns of the selected areas. **f, g** AC-HAADF-STEM image of NiFeLa. **h, i** Integrated pixel intensities of the NiFeLa taken from the orange and rose lines in **g**, respectively

**Fig. 2:**
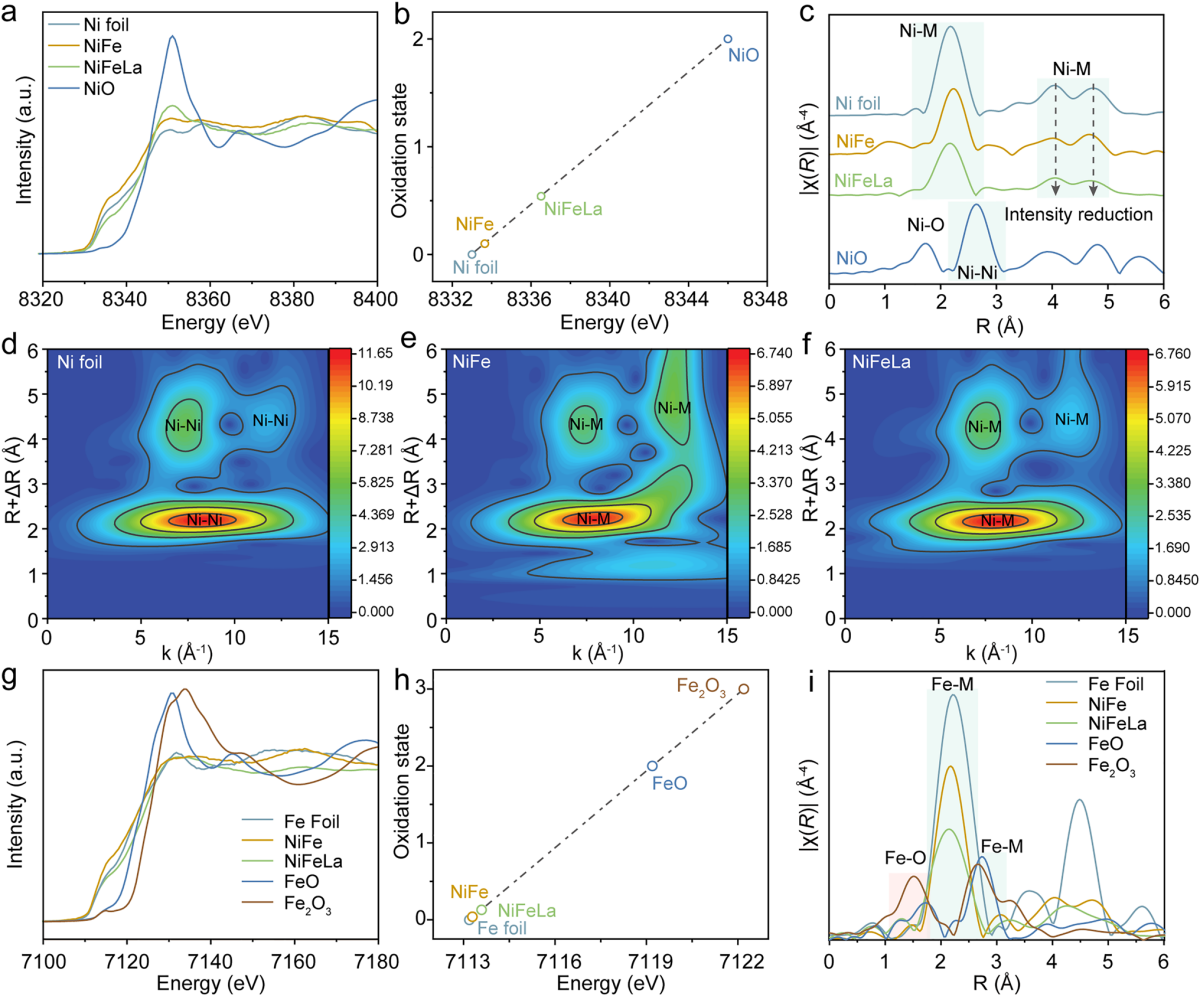
**a** XANES spectra at the Ni K-edge of NiFe, NiFeLa, Ni foil and NiO. **b** Oxidation state of various Ni species obtained from Ni K-edge XANES. **c** FT-EXAFS of NiFe, NiFeLa, Ni foil and NiO. **d-f** Wavelet transform of Ni K-edge EXAFS data of Ni foil, NiFe and NiFeLa. **g** Ni 2*p* XPS spectra of NiFe and NiFeLa. **h** Fe 2*p* XPS spectra of NiFe and NiFeLa. **i** La 3*d* XPS spectra of NiFeLa

We first develop a straightforward one-step method using in situ electrodeposition technique to synthesize the ternary NiFeM (M: 4*d*-Mo/5*d*-La) catalysts. Based on the inductively coupled plasma optical emission spectroscopy (ICP-OES) results present in Fig. S1, the doping levels of Mo and La in NiFeMo and NiFeLa are consistent, ruling out the influence of different levels of Mo and La on the results. As evidenced by X-ray diffraction (XRD), the diffraction peaks at 44.4°, 51.6°, and 76.2° can be assigned to the (111), (200), and (220) planes of NiFe alloy, respectively (PDF#38–0419) (Fig. 1a). The introduction of La and Mo atoms results in typical varying degrees of variations in XRD peak intensity and peak broadening of the NiFe sample, suggesting the construction of asymmetric M-NiFe units. Additionally, scanning electron microscopy (SEM) images reveal that the NiFe-based samples (NiFe, NiFeMo, and NiFeLa) are all uniformly grown on the carbon cloth (CC) with nanoparticle morphologies (Figs. S2–S4). HAADF-STEM image and energy-dispersive X-ray (EDS) for NiFeLa with elemental mapping images demonstrate the uniform distribution of Ni, Fe, and La elements (Figs. 1b and S5). High-Resolution Transmission Electron Microscope (HRTEM) image and the fast Fourier transform (FFT) pattern indicate the good crystallinity of NiFe alloy (Fig. S6). The TEM images in Figs. 1c and S7 demonstrate the NiFeMo exhibits a polycrystalline structure with part of amorphous areas. The spacing of 2.08 Å is recognized as the (111) plane of NiFeMo. Based on the HRTEM images of NiFeLa in Fig. 2d, it is evident that there exist numerous grain boundaries among the NiFeLa particles, as well as localized ordered regions (marked by the blue dashed lines) with a diameter smaller than 5 nm. In contrast, NiFeMo exhibits compact nanocrystals as well as localized regions with order exceeding 10 nm, align with the XRD results. In addition, the (111) plane of NiFeLa has a larger spacing (2.10 Å), indicating that the introduction of La element leads to more significant lattice distortion (Figs. 2e and S8) [33]. The fine structure of NiFeLa is studied using the HAADF-STEM with aberration correction (AC-HAADF-STEM). In the high-resolution AC-HAADF-STEM image of NiFeLa, the presence of grain boundaries (Fig. 2f, g) and the variation of Ni-M (*M* = Ni, Fe or La) distance (Fig. 2h, i) further confirm the successful incorporation of La and the distorted structure of NiFeLa [34]. And La atoms are located in the same column as the Ni/Fe atoms, indicating that La replaces the Ni/Fe in the lattice of NiFe. Therefore, it can be considered that this one-step method can effectively induce the dispersion of La atoms within NiFe, disrupting the symmetry of Ni–Fe units and assisting the construction of gradient 3*d*-5*d* orbital coupled Ni/Fe-M asymmetric geometric structure.

X-ray absorption spectroscopy (XAS) is utilized to investigate modifications in both the structural and electronic properties. The X-ray absorption near-edge structure spectra (XANES) at Ni K-edge shown in Fig. 2a, b demonstrate that the absorption threshold position of Ni atom in NiFeLa is higher than that of Ni foil reference and NiFe alloy, suggesting the valence state of Ni increases after the introduction of La [35]. In addition, the Ni K-edge Fourier transforms extended X-ray absorption fine structure (FT-EXAFS) spectra of NiFeLa show three peaks located at about 2.48, 4.05, and 4.7 Å, which can be assigned to the Ni-M bonds (Figs. 2c and S9) [17, 36]. Compared to NiFe, the NiFeLa displays a significant decrease in the peaks’ intensity at Ni-M distance, further suggesting the lattice distortion and asymmetric La-Ni/Fe units [37]. The wavelet transform (WT) analysis further offers information in both R- and K-space information of those samples, revealing the overlapping contributions in the radial distance. Additionally, the local coordination environment of Ni is explored using extended X-ray absorption fine structure spectra (EXAFS) along with their respective wavelet-transformed (WT) contour plots (Fig. 2d–f). The relatively longer Ni-M bond located at ~ 7.8 Å^−1^ can be observed in the pattern of NiFeLa, further proving the lattice distortion of NiFeLa [38, 39]. The results from XANES of Fe K-edge for NiFeLa and NiFe shown in Fig. 2g, h, demonstrate the increased valence state of Fe after the introduction of La. Compared with the FT-EXAFS spectra of NiFe, the peak intensity at Fe-M bonds in NiFeLa is notably reduced, indicating the presence of lattice distortion and asymmetric La-Ni/Fe units, which aligns with the FT-EXAFS spectra of the Ni K-edge (Figs. 2i and S10). The relatively longer Fe-M bond observed in the WT contour plots of NiFeLa further proving the asymmetric La-Ni/Fe units (Fig. S11). In addition, the fitting curves indicate that as the introduction of La element, the Ni-M (*M* = Ni, Fe, La) and Fe-M bonds move to the structures with low coordination numbers (Tables S1 and S2). The surface chemical states of the NiFe alloy and NiFeLa alloy are further investigated by X-ray photoelectron spectroscopy (XPS) (Fig. S12). Notably, the positive shifts of Ni 2*p* and Fe 2*p* of NiFeLa compared to those of NiFe can be observed, agreeing well with the XAS results [36].

### Electrocatalytic OER Performances

First, the electrocatalytic performance of NiFeLa catalysts with different La ratios is assessed using a conventional three-electrode setup in 1 M KOH electrolyte solution. The content of La in NiFeLaX (*X* = 0.25, 0.5, 1, 1.5) determined by ICP-OES are shown in Fig. S13 and Table S3. As depicted in Fig. S14a, the LSV curves reveal that the NiFeLa1.5 exhibits remarkable OER activity, with an overpotential of 185 mV to achieve the current density of 10 mA cm^−2^, which is much lower than those of NiFe (244 mV), NiFeLa0.25 (201 mV). While there is no significant difference in the overpotential of NiFeLa0.5 (190 mV) and NiFeLa1 (187 mV) compared to that of NiFeLa1.5. Furthermore, as shown in Fig. S14b, NiFeLa0.5, NiFeLa1, and NiFeLa1.5 exhibit the same Tafel slope of 34.3 mV dec^−1^. Additionally, the long-term stability of the synthesized NiFeLaX (*X* = 0.25, 0.5, 1.5) is further examined within 1 M KOH (Fig. S14c). The chronopotentiometric tests indicate that NiFeLa0.5 exhibits the most outstanding OER stability, maintaining the current density of 1A cm^–2^ for > 100 h, which is longer than those of NiFe, NiFeLa0.25, NiFeLa1 and NiFeLa1.5. As depicted in Fig. S14d, the NiFeLa0.5 possesses the most suitable OER activity and stability. Therefore, we choose NiFeLa0.5 for further comparison and NiFeLa refers to NiFeLa0.5 in the whole text.

**Fig. 3:**
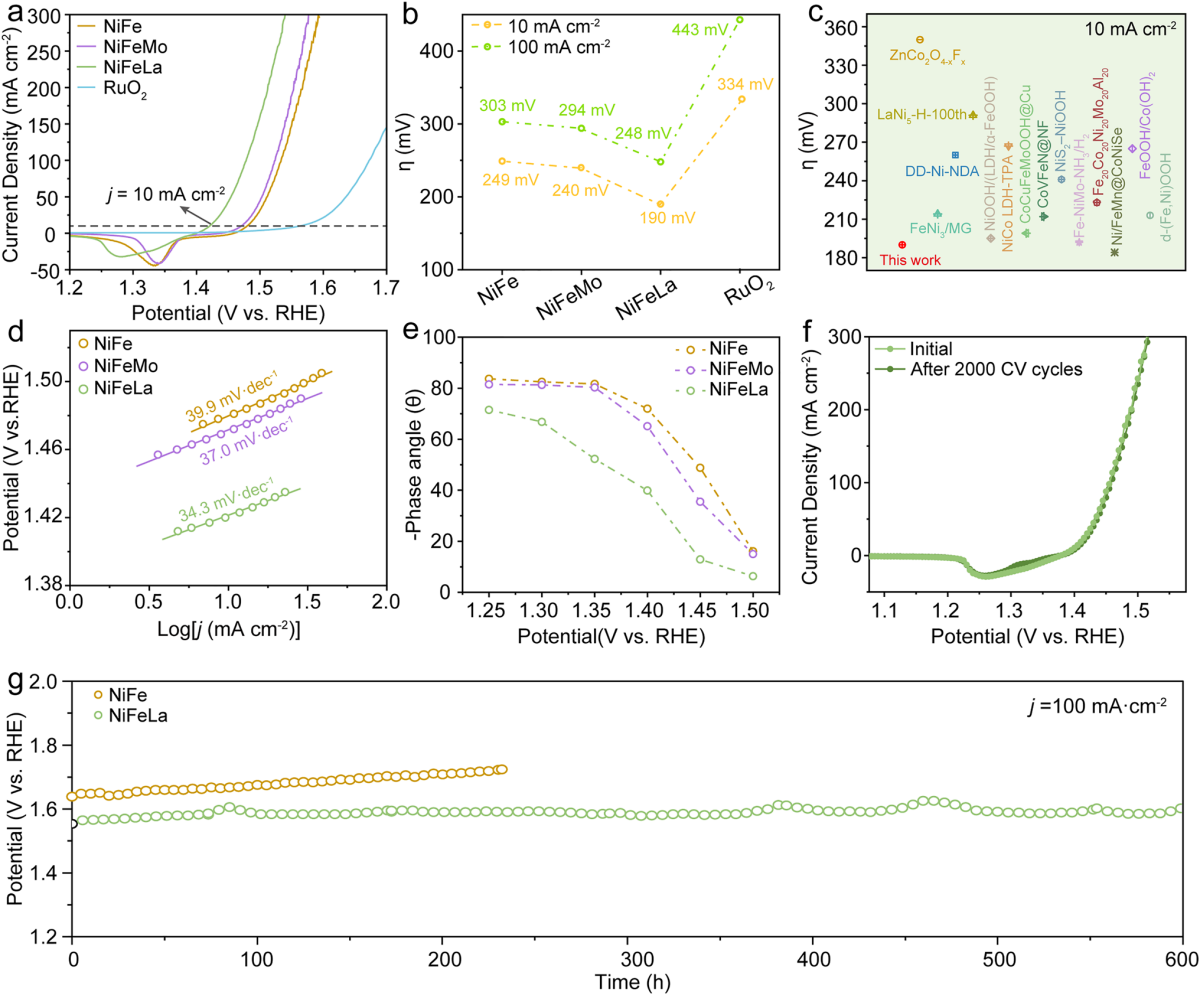
**a** LSV curves of NiFe, NiFeMo, NiFeLa and commercial RuO_2_ in 1 M KOH solution at oxygen-saturated atmosphere. **b** Comparison of the overpotential at the current density of 10 and 100 mA cm^−2^ according to LSV curves. **c** Comparison of the overpotential at the current density of 10 mA cm^−2^ of NiFeLa with recently reported transition metal-based OER electrocatalysts in alkaline electrolyte. **d** Corresponding Tafel plots according to the LSV curves in Fig. 3a. **e** Summarized phase peak angles of NiFe, NiFeMo, and NiFeLa at 1.25–1.50 V. **f** LSV curves of NiFeLa before and after 2000 cycles CV. **g** Chronopotentiometric curves of NiFe and NiFeLa at current density of 100 mA cm^−2^

The electrocatalytic performances of NiFeLa, NiFeMo and commercial RuO_2_ are further evaluated in 1 M KOH electrolyte by using a standard three-electrode system. Figure 3a, b shows that NiFeLa displays the best OER activity, with the overpotential of 190 and 248 mV to achieve the current density of 10 and 100 mA cm^–2^, respectively, which are significantly lower than those of NiFe (249 and 303 mV), NiFeMo (240 and 294 mV) and commercial RuO_2_ (334 and 443 mV). It is noteworthy that the catalytic activity of NiFeLa surpasses most of the reported transition metal-based alkaline electrocatalysts (Fig. 3c and Table S4). The excellent OER activity of NiFeLa is further evidenced by the lowest Tafel slope, suggesting its faster kinetics during the OER process (Fig. 3d) [40]. Furthermore, the electrocatalytic kinetics during the OER process are probed using in situ electrochemical impedance spectroscopy (EIS) measurements, conducted under different applied voltages. As shown in Fig. S15, NiFeLa displays the most diminutive Nyquist semicircle diameter, suggesting the most minimal R_ct_ experienced throughout the OER process. In the Bode phase plots, the highest phase angle peaks observed in the high-frequency and low-frequency regions correspond to the inherent electron conduction of the electrocatalyst and the charge transfer occurring at the interface between the electrolyte and the catalyst, respectively [41]. As shown in Figs. S16–S18, the samples display higher phase peaks at low-frequency regions (~ 0.01–10 Hz) compared to high-frequency regions (~ 100–10,000 Hz), unveiling that the charge transfer is predominantly constrained by the resistance at the electrolyte-catalyst interface. Upon raising the applied potentials from 1.30 to 1.55 V (vs. RHE), the phase angles at low-frequency for NiFeLa demonstrate an accelerated decline trend compared with other catalysts, suggesting La dopping facilitates the electron transfer at electrolyte-catalyst interface, leading to superior OER kinetics (Fig. 3e) [42]. In addition, the electrochemically active surface area (ECSA) was conducted to evaluate the electrochemical features of those electrocatalysts by employing double-layer capacitance (*C*_dl_) via CV (Fig. S19). As shown in Fig. S19d, NiFeLa displays the highest *C*_dl_ of 13.4 mF cm^−2^, indicating the most active site exposure. Additionally, as shown in Table S5, the specific activity, represented by the current density normalized by the electrochemical surface area, further suggests the remarkable catalytic activity of NiFeLa. In addition, to understand the interaction between La and Ni/Fe, in situ ultraviolet (UV) spectra at different potentials are carried out (Fig. S20). The spectra are collected from open circuits potential (OCP) to 1.6 V with an interval of 0.05 V. As shown in Fig. S20a, the spectra of NiFe display a peak appearing at around 350 nm when the potential at 1.4 V and above, which can be attributed to the signal of *OOH-NiFe. The peak located at ~ 450 nm appears from 1.45 V and remains unchanged when the voltage exceeds 1.60 V, identifying the generation of *OO-NiFe [1, 43]. Simultaneously, the spectra of NiFeMo in Fig. S20b demonstrate distinctive peaks at ~ 350 nm when the potential at 1.4 V and above, which can be attributed to the signal of *OOH-NiFeMo. And a broad peak range 400 to 700 nm emerges and remains constant in the OER relevant potential region (≥ 1.45 V), corresponding to *OO-NiFeMo. While prominent peaks can be observed at ~ 350 nm (*OOH-NiFeLa) with a voltage of 1.35 V for NiFeLa (Fig. S20c). It is worth noting that unique peak around ~ 600 nm is observable in the OER-relevant potential range (≥ 1.40 V), corresponding to the accumulation of *OO-NiFeLa. Based on the above results, it can be inferred that NiFeLa is more prone to losing electrons to form *OOH and *OO species at the lowest potential, thereby exhibiting higher OER activity. The electrocatalytic stabilities of NiFeLa and NiFe during OER are also investigated. As shown in Fig. 3f, NiFeLa exhibits an unabated activity similar to its initial state after 2000 cycles of CV. The chronopotentiometric test indicates that the current density of 100 mA cm^–2^ shows negligible degradation for > 600 h, while the stability of the NiFe catalyst decreases significantly (Fig. 3g). Besides, the XRD and XPS spectra of NiFeLa after OER test shown in Figs. S21 and S22 indicate that the NiFeLa catalyst can effectively maintain the structural stability throughout the OER process.

### Mechanism Exploration

**Fig. 4:**
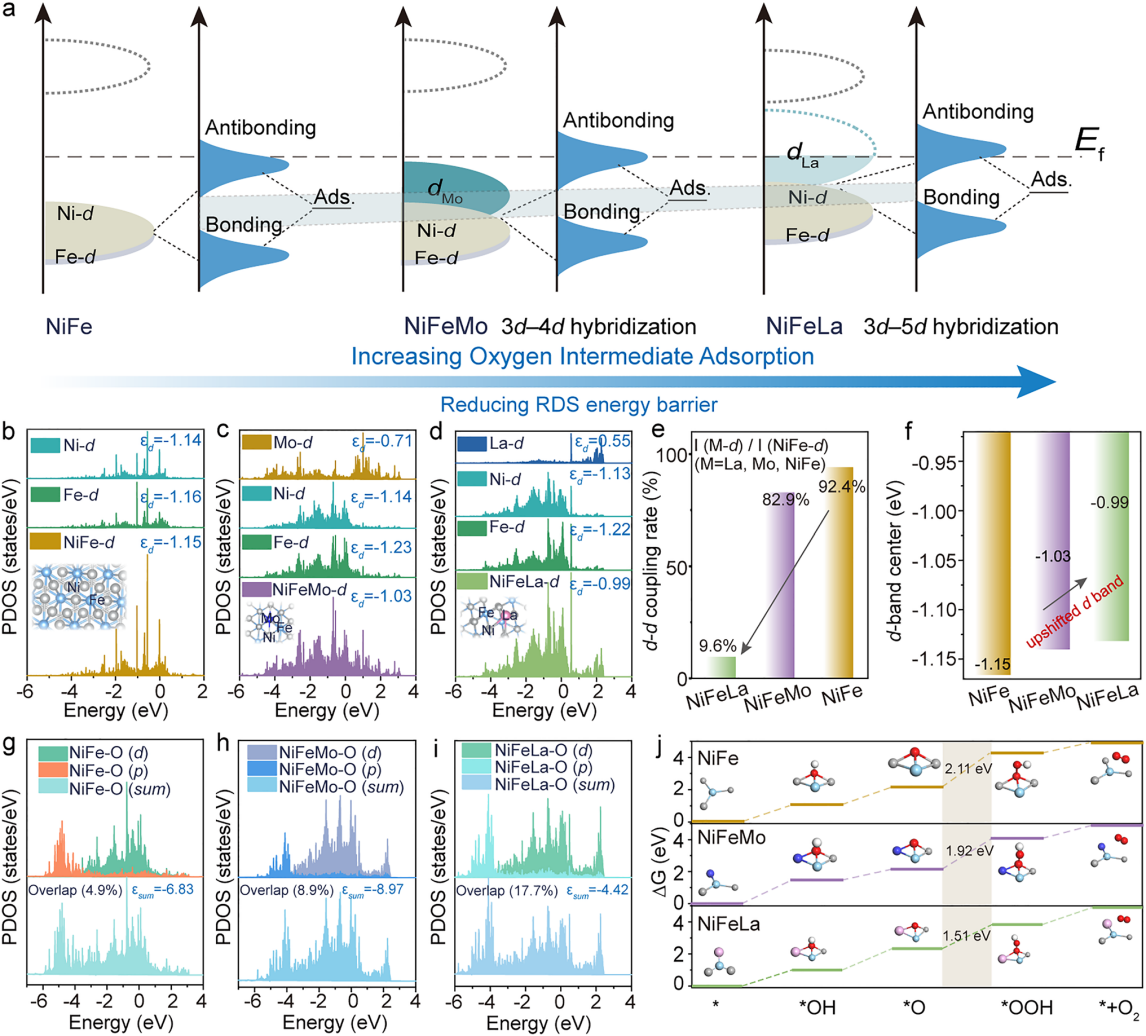
**a** Schematic illustration of bond formation between the reaction surface and the adsorbate (Ads.). The partial density of states (PDOS) analysis of **b** NiFe, **c** NiFeMo and **d** NiFeLa. **e** *d*-*d* coupling rate of M (Ni, Mo, La) and Ni/Fe in NiFeLa, NiFeMo, and NiFe. **f** *d*-band center of adsorbed atoms in the NiFe, NiFeMo and NiFeLa. The PDOS analysis of **g** NiFe-O, **h** NiFeMo-O and **i** NiFeLa-O. **j** Free energy diagram of OER on NiFe, NiFeMo, NiFeLa

To in-depth explore the origin of the enhanced OER activity by NiFeLa, the density functional theory with Coulomb interactions (DFT + U) calculations were probed (Figs. S23–S26). We firstly analyzed the adsorption energy of oxygen intermediates on different adsorption sites. The results indicate that it is energetically favorable for the doped La/Mo atoms to replace Ni atoms in NiFe and the oxygen intermediates absorbed on hollow sites, as shown in Figs. S24–S26. Furthermore, it is well known that optimizing the adsorption/desorption of oxygen intermediates is crucial for improving the OER performance of NiFe catalysts in alkaline electrolytes. Thus, the electronic interaction could be considered as the coupling between the adsorbate states and the transition-metal *d* states, which gives rise to the formation of separated bonding and antibonding states. The bonding states are generally fully filled because they are far below the Fermi level (*E*_*f*_), while the electron filling of the antibonding states depends on these energy states relative to the Fermi level (i.e., *d* band center) and contributes to the bond strength (Fig. 4a) [44–46]. The *d*-orbital density of states (DOS) of different sites in NiFe/NiFeMo/NiFeLa is further investigated to probe the adsorption strength between the oxygen intermediates and NiFe before and after Mo/La doping (Fig. 4b–d). Specially, benifiting from the mismatch of orbital energy in NiFeLa than those of NiFe and NiFeMo (Fig. 4e), the* d*-band center of adsorbed atoms in the NiFeLa (−0.99 eV) is higher than those in NiFe (−1.15 eV) and NiFeMo (-1.03 eV) (Fig. 4f). Therefore, compared to NiFe and NiFeMo, the antibonding electron of NiFeLa decreases with elevation of antibonding orbitals, resulting in enhanced adsorption strength between oxygen intermediates and NiFeLa. Moreover, as shown in Fig. S27, the La and Mo atoms would lose electrons (negative value), while the NiFe atoms would obtain electrons (positive value). Notably, the charge loss in NiFeLa is much higher than those in NiFe and NiFeMo, further indicating the facilitating the adsorption of adsorbates [47]. Consequently, the adsorption behavior of *OOH-NiFeM in different NiFeM is further calculated. And the enhanced adsorption strength of *OOH in NiFeLa is observed than NiFeMo/NiFe, as indicated by the decreased antibonding electron in NiFeLa-O bond and increased orbital coupling between O-*p* and NiFeLa-*d* (Fig. 4g–i), and the strongest M–O in *OOH-NiFeLa (Fig. S28) [48–50], as well as the largest electron overlaps in *OOH (Fig. S29) and the highest electronic local functions (Fig. S30). As expected, the enhanced adsorption strength of *OOH caused by mismatched 5*d* orbital coupling in NiFeLa is favorable to decrease the rate-determining step (RDS) (*O → *OOH) (Figs. 4j and S31).

To further verify the relationship between different La contents and OER activity and stability, we also conducted DFT calculations with various La doping (Figs. S32-S35). As expected, compared to NiFeLa-1 (with a La content ~ 5%), the *d*-band center of the adsorbed atoms in NiFeLa-2 and NiFeLa-3 catalysts rises with the increased La doping (Fig. S36), thereby gradually enhancing the adsorption of oxygen intermediates (Fig. S37a). Specifically, the energy barriers of PDS remained relatively unchanged with different La doping. However, as the amount of La increases, the surface energy gradually increased (Fig. S37b), indicating a decreased stability with increased La doping, which is consistent with the electrochemical results.

### Electrochemical Evaluation of AEMWE Device

**Fig. 5:**
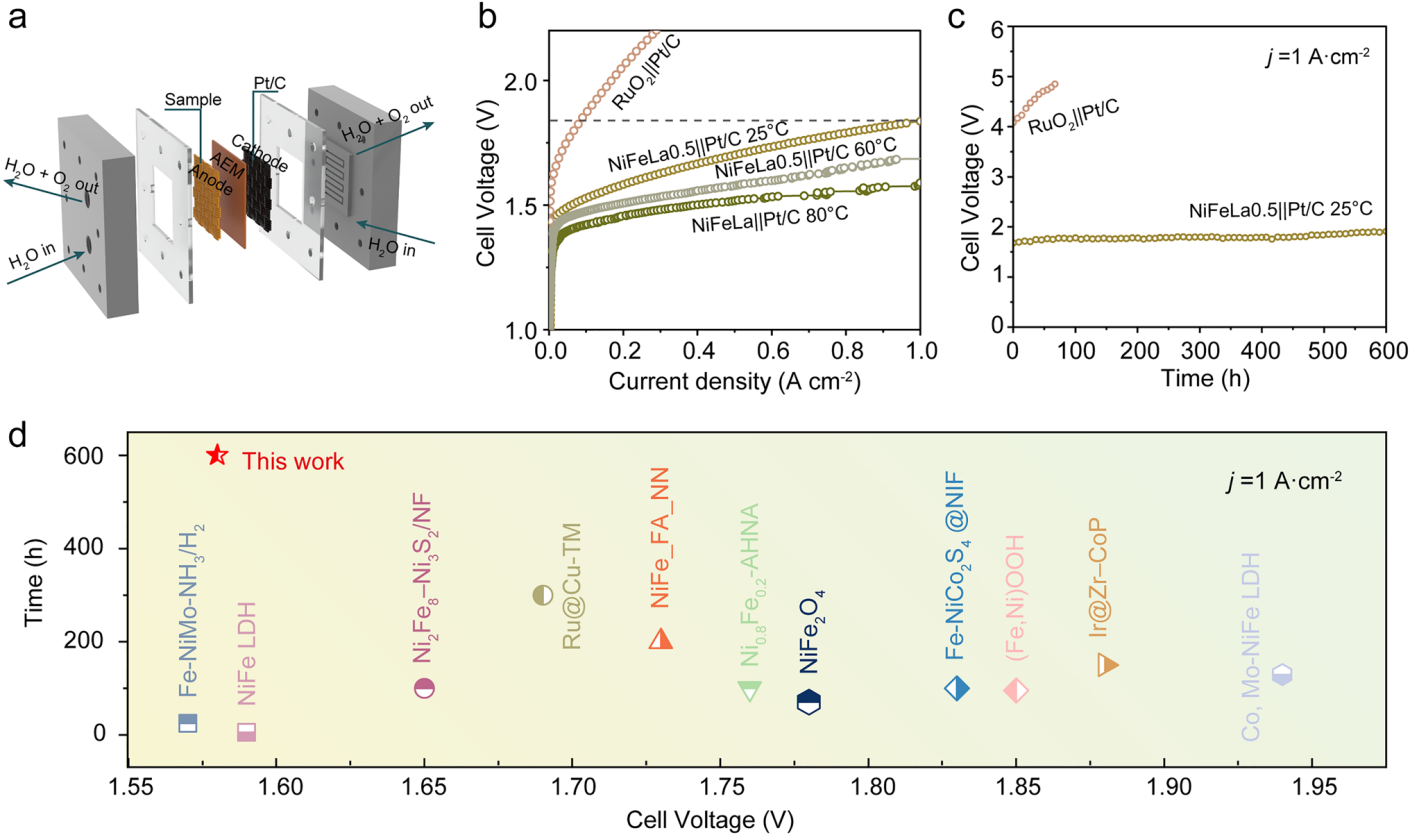
**a** Schematic illustration of the designed AEM water electrolyzer. **b** LSV curves of AEM water electrolyzer for NiFeLa//Pt/C at 25, 60, and 80 °C, respectively, and commerical RuO_2_||Pt/C cells at 25 °C. **c** Durability cell voltage–time plots for the AEM water electrolyzer at a constant current density of 1 A cm^−2^ for 600 h. **d** Comparison the current density of AEM water electrolyzer for the NiFeLa||Pt/C cell and the reported AEM water electrolyzer cells at the current density of 1 A cm^−2^ in 1 M KOH

Encouraged by the overall high activity and stability of NiFeLa towards OER, we assemble a single cell using the prepared catalyst as the anode to evaluate its performance in a real AEMWE device (Figs. 5a and S38a). Excitingly, cell voltages of only 1.7/1.83, 1.58/1.68, and 1.52/1.58 V are needed for NiFeLa to reach the current densities of 0.5 and 1 A cm^−2^ at 25, 60, and 80 °C, respectively, much superior to that obtained with commercial RuO_2_ (2.05 V@0.2 A cm^−2^) and NiFe (1.82/2.08, 1.72/1.93, and 1.68/1.84 V) (Figs. 5b and S39). The steady-state polarization curves operated at 25, 60, and 80 °C, demonstrate that the performance of AEMWE can be greatly improved with the utilization of NiFeLa. In addition, the Faraday efficiencies of O_2_ production are also measured, which fit well with the theoretical yields with the molar ratio of O_2_, indicating a Faraday efficiency of almost 99% (Fig. S38b–d). Furthermore, as shown in Fig. 5c, the NiFeLa cell demonstrates well-maintained AEMWE performance after running for over 600 h under 1 A cm^−2^ with no significant decrease, surpassing commercial RuO_2_ and most of the reported transition metal-based catalysts, describing the remarkable OER performance of NiFeLa under practical operation conditions (Fig. 5d and Table S6).

## Conclusions

In summary, we present a practical concept by constructing asymmetric M-NiFe units to regulate the *d*-orbital and electronic structures of NiFe-based catalysts. The 5*d*-introduced NiFeLa shows remarkable activity and stablility toward OER, as well as long-term operation in AEMWE devices. Experimental results and DFT calculations indicate that the introduction of La atoms into NiFe could disrupt the symmetry of the Ni–Fe units, optimize the *d*-*p* orbital hybridization between the metal sites on the surface of the catalyst and oxygen-containing intermediates, thereby reducing the RDS energy barrier and boosting the OER performance. This work offers a feasible solution for the practical application of NiFe-based catalysts in commercial AEMWE systems through the electronic regulation strategy induced by *d*-orbital hybridization.

## Supplementary Information

Below is the link to the electronic supplementary material.

Supplementary file1 (DOCX 6651 KB)
