# Supplementary material for: Boosting Oxygen Evolution Reaction Performance on NiFe-Based Catalysts Through d-Orbital Hybridization
Source: Nanomicro Lett. 2024 Sep 26;17:11. doi: 10.1007/s40820-024-01528-9 (PMC11427650; doi:10.1007/s40820-024-01528-9)
Supplement: Supplementary file 1 — Supplementary file1 (DOCX 6651 KB) [file 40820_2024_1528_MOESM1_ESM.docx]

Supporting Information for

Boosting Oxygen Evolution Reaction Performance on NiFe-based Catalysts through *d*-Orbital Hybridization

Xing Wang^1,#^, Wei Pi ^1,#^, Sheng Hu ^1^, Haifeng Bao ^1,^*, Na Yao ^1,^*, and Wei Luo ^2,^*

^1^State Key Laboratory of New Textile Materials and Advanced Processing Technology, Key Laboratory of New Textile Materials and Applications of Hubei Province, School of Materials Science and Engineering, Wuhan Textile University, Wuhan 430200, P. R. China

^2^ College of Chemistry and Molecular Sciences, Wuhan University, Wuhan 430072, P. R. China

^#^ Xing Wang and Wei Pi contributed equally to the work.

* Corresponding authors. E-mail: [baohaifeng@wtu.edu.cn](mailto:baohaifeng@wtu.edu.cn) (Haifeng Bao), [yaona@wtu.edu.cn](mailto:yaona@wtu.edu.cn) (Na Yao), [wluo@whu.edu.cn](mailto:wluo@whu.edu.cn) (Wei Luo)

**S1 Materials Characterization**

X-ray diffraction (XRD) patterns were collected on an Empyrean PANalytical diffractometer with Cu-Kα irradiation (40 kV, 40 mA, λ = 1.5418 Å) at a scanning rate of 5° min^-1^. The morphologies and sizes of the samples were observed by scanning electron microscopy (SEM, SIGMA, ZEISS) equipped with an energy dispersive X-ray detector (EDX), and Tecnai G20 U-Twin transmission electron microscope (TEM) at an acceleration voltage of 200 kV. Thermo Scientific K-Alpha^+^ instrument was used to collect the X-ray photoelectron spectrometry (XPS) information. The calibration peak data was based on the C 1s peak at 284.8 eV. The chemical compositions were determined by inductively coupled plasma optical emission spectroscopy (ICP-OES, Skyray Instrument ICP2060T). Diffuse reflectance spectra were measured using a Cary 5000 spectrophotometer fitted with an integrating sphere attachment from 200-800 nm with BaSO_4_ as the reference.

To perform X-ray absorption spectroscopy (XAS) analysis, we prepared the test samples on titanium foil and then brushed them to obtain the powders. The resulting powders were uniformly painted on plastic radiation-resistant Scotch tape (Kapton tape, the duct tape of the synchrotron) with a length of 10 cm and width of 1 cm. To achieve compact particle distribution on Scotch tape without pinholes, after the large particles were shaken off, the sample tape was cut into 10 small pieces, which were stacked layer by layer on another Scotch tape and covered with Piece of scotch tape. Ni K-edge X-ray absorption spectra (XAS) were obtained on the 1W1B beamline of the Beijing synchrotron radiation facility, People’s Republic of China, operated at 200 mA and 2.5 GeV. Ni foil was used as the reference sample, and all samples were measured in transmission mode. The XAFS data were analyzed using the software package Demeter. The spectra were normalized using Athena first, and then shell fittings were performed with Artemis. The χ(k) function was Fourier transformed (FT) using k^3^ weighting, and all fittings were performed in R-space.

**S2 Electrochemical Characterizations**

The OER catalytic performance was evaluated in a three-electrode system on a CHI760e electrochemical workstation. Briefly, NiFeM was used as working electrodes, while carbon rod and Hg/HgO electrode were used as the counter electrode and reference electrode, respectively. Linear sweep voltammetry (LSV) curves were measured in 1 M KOH solution at a scan rate of 5 mV/s with 85% inner resistance (iR) compensation. Stability curves were measured by Chronopotentiometry without iR compensation. Electrochemical impedance spectroscopy (EIS) was taken over a frequency from 100 KHz to 0.01 Hz with the input voltage at 10 mA·cm^-2^ current density. Electrochemically active surface areas (ECSA) were measured by cyclic voltammetry (CV) in the potential window from 0 to 0.2 V vs. Hg/HgO under scan rates of 20, 40, 60, 80, 100, 120, 140, 160, 180, and 200 mV s^−1^. The current density at 0.1 V vs. Hg/HgO was used for the calculation of ECSA. The electrical double-layer capacitance (Cdl) was calculated by the equation Cdl = (j_a_-j_c_)/2ν, where j_a_ and j_c_ are the anodic current density and cathodic current density, respectively, and ν is the scan rate. Thus, Cdl is the slope of the linear relationship between (j_a_-j_c_)/2 and scan rates. The ECSA can be calculated by ECSA = Cdl/Cs. The Cs value of 0.04 mF cm^-2^ was acquired in alkaline solutions without taking the used material and measurement conditions into account.[1] All the measured potentials versus Hg/HgO were converted to a reversible hydrogen electrode (RHE) by the Nernst equation of E_RHE_ = E_Hg/HgO_ + 0.098 + 0.059 × pH. All electrochemical measurements were performed in 1 M KOH if without otherwise specified.

**S3 DFT Calculations**

DFT calculations were performed using the VASP with the Perdew−Burke−Ernzerhof (PBE) level and project-augmented wave (PAW) method [2]. The project-augmented wave (PAW) method was used to represent the core−valence electron interaction. A 3×3×1 Monkhorst-Pack grid of k-points was used for the Brillouin zone integration. The valence electronic states were expanded in plane-wave basis sets with an energy cutoff at 450 eV. Gaussian smearing of 0.05 eV was applied during the geometry optimization. The convergence criteria for the iteration in the self-consistent field (SCF) were set at 10-5 eV, and the residual force for optimizing atom positions was less than 0.02 eV/Å. The effective U-J values were fixed at 4.2 eV for Fe, 3.8 eV for Ni, 8.6 eV for Mo and 6.0 eV for La.

The OER performance indicator, overpotential η, is readily available as the OER proposed to adopt the following reaction pathway:

(1) *+H_2_O → *OH + (H^+^ + e^−^) ΔG_1_

(2) *OH → *O + (H^+^ + e^−^) ΔG_2_

(3) *O + H_2_O → *OOH + (H^+^ + e^−^) ΔG_3_

(4) *OOH → *+O_2_ + (H^+^ + e^−^) ΔG_4_

where * presents the active site for the reaction, ΔG_1_, ΔG_2_, ΔG_3_ and ΔG_4_ represent the free energy changes of each step, and

(5) η = max (ΔG_1_, ΔG_2_, ΔG_3_, ΔG_4_) – 1.23

The Gibbs free energy change (ΔG) is defined as:

ΔG = ΔE + ΔZPE – TΔS

where ΔE is the reaction energy of the adsorbed reactant and product molecules, ΔZPE stands for the zero-point energy correction, T equals to 298.15 K and ΔS represents the change of entropy.

ΔE is calculated on basis of the equation:

ΔE = E_adsorbent@slab_ – E_slab_ – E_adsorbent_

Where E_adsorbent@slab_, E_slab_ and E_adsorbent_ represent the energies of substrate with reaction intermediates adsorbed, substrate and reaction intermediates, respectively.

**Supplementary Figures and Tables**


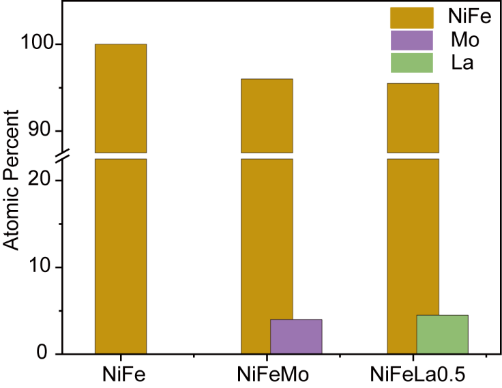


**Fig. S1** The content of NiFe, Mo and La in NiFe, NiFeMo and NiFeLa were determined by ICP-OES


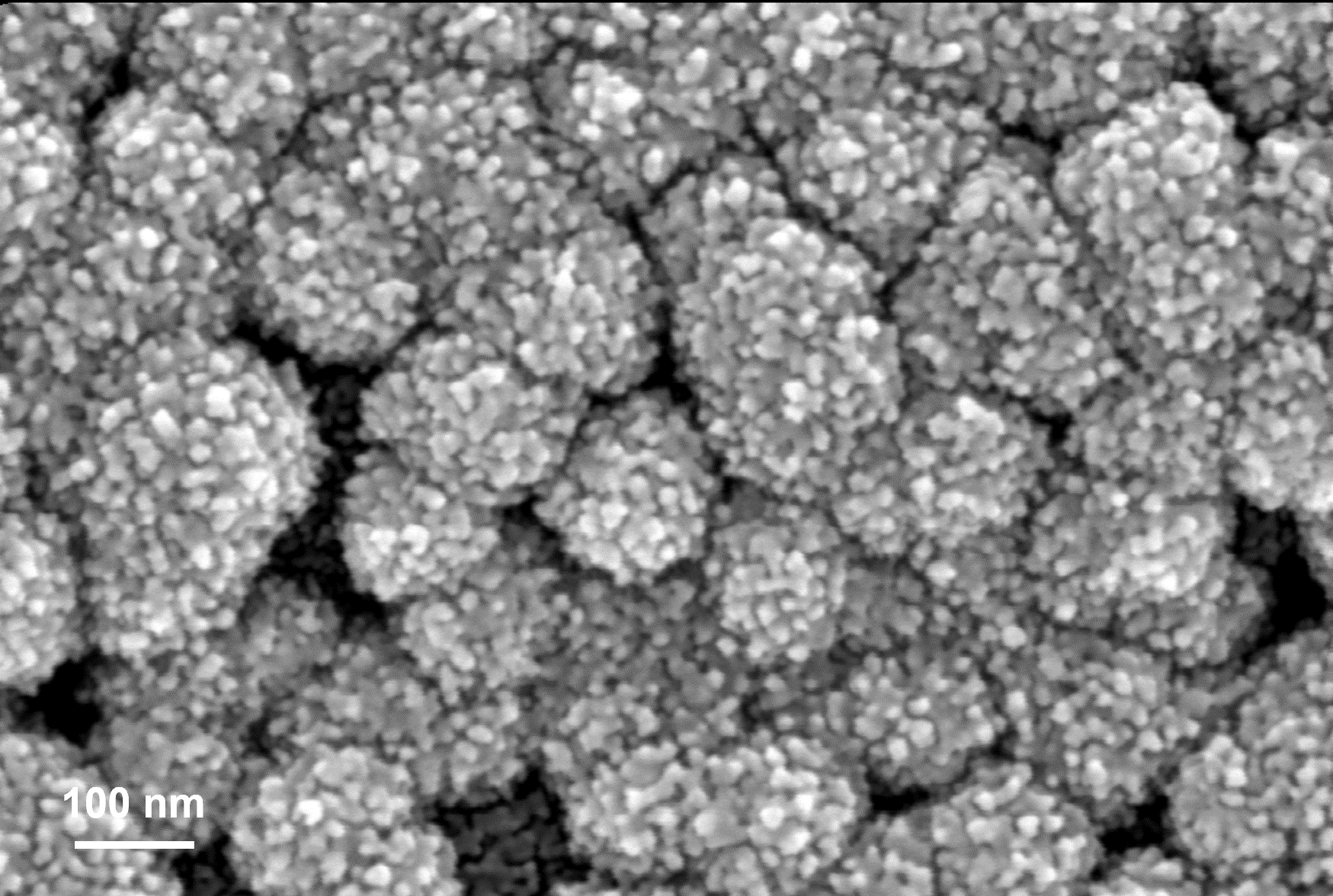


**Fig. S2** SEM image of prepared NiFe alloy


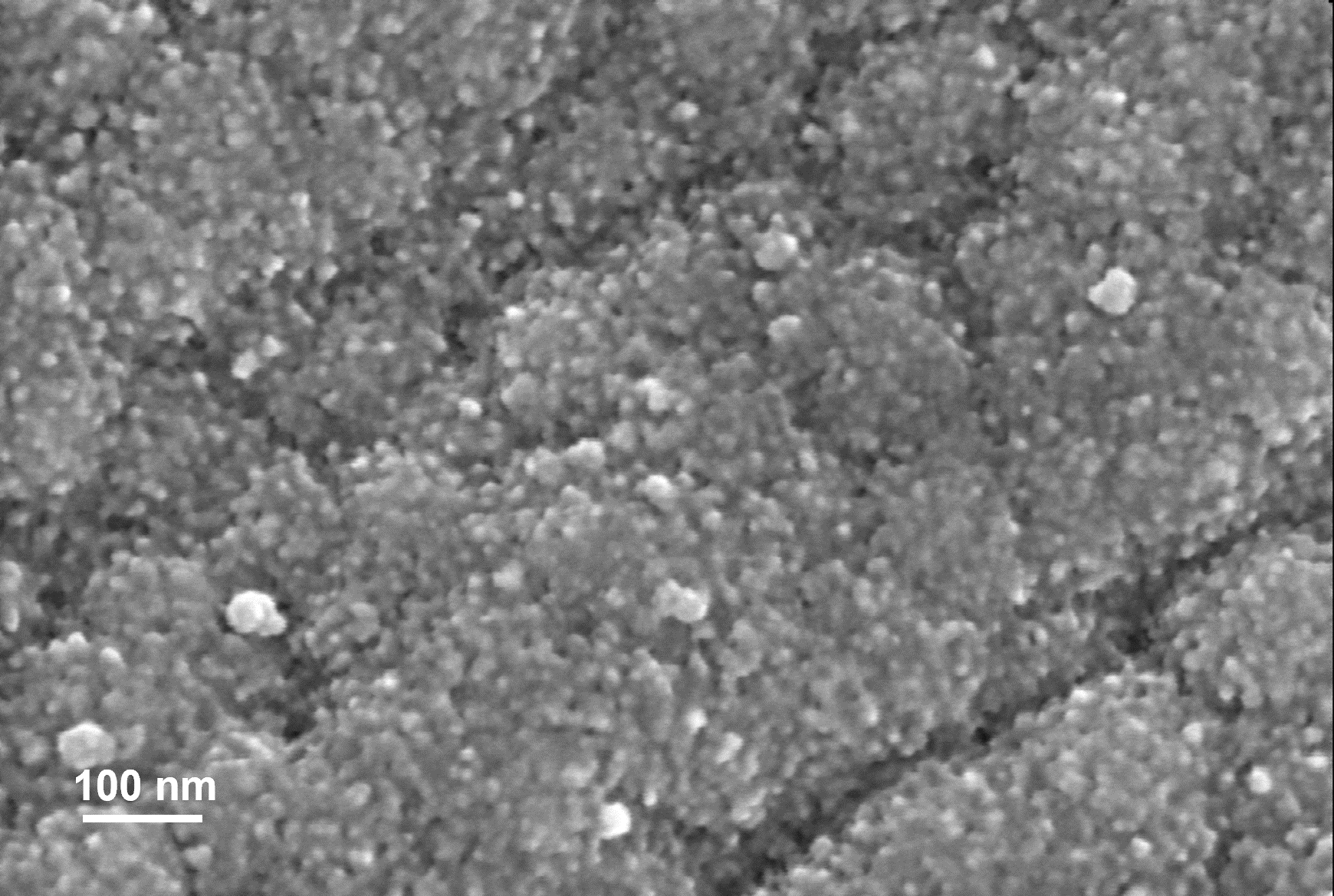


**Fig. S3** SEM image of prepared NiFeMo alloy


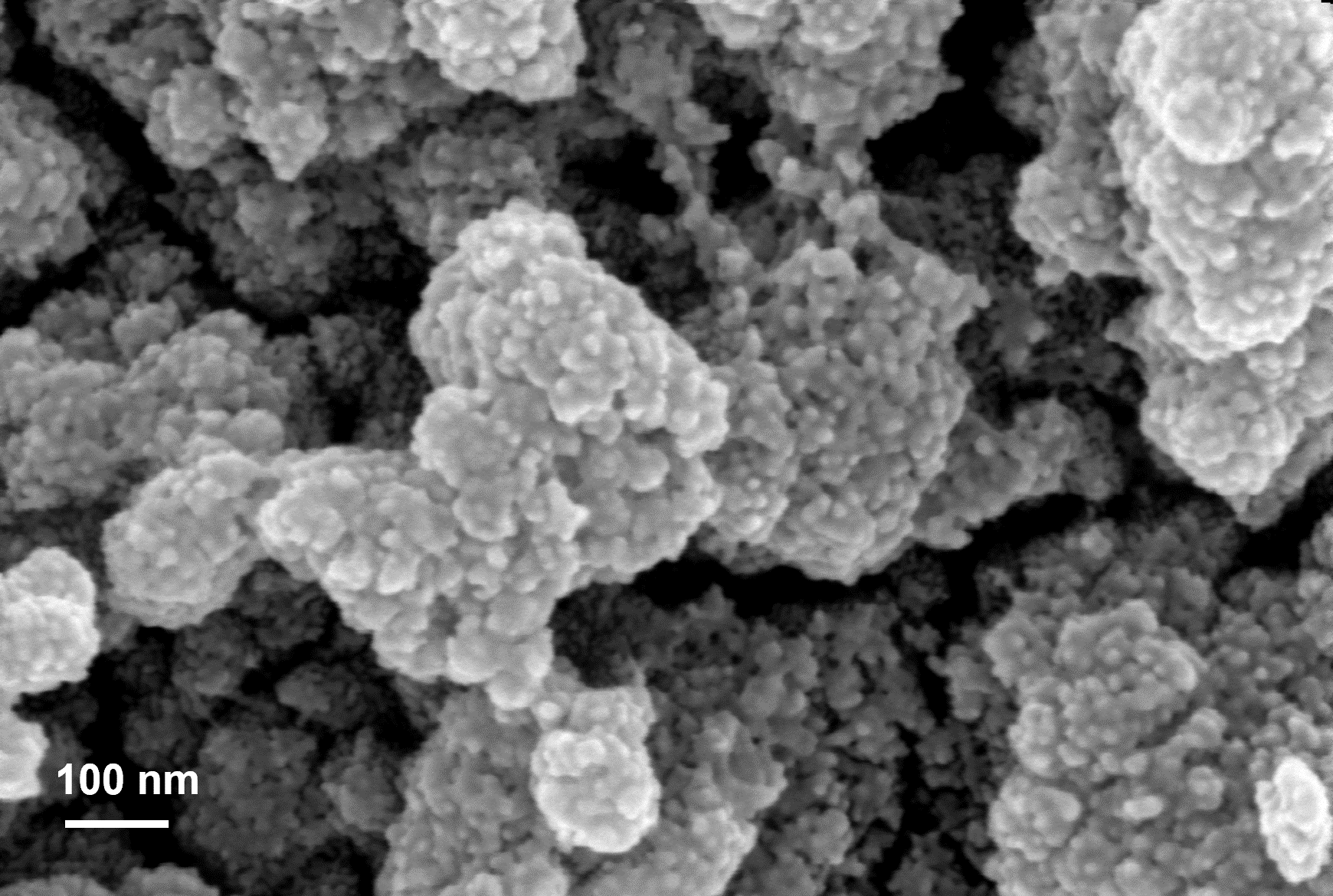


**Fig. S4** SEM image of prepared NiFeLa alloy


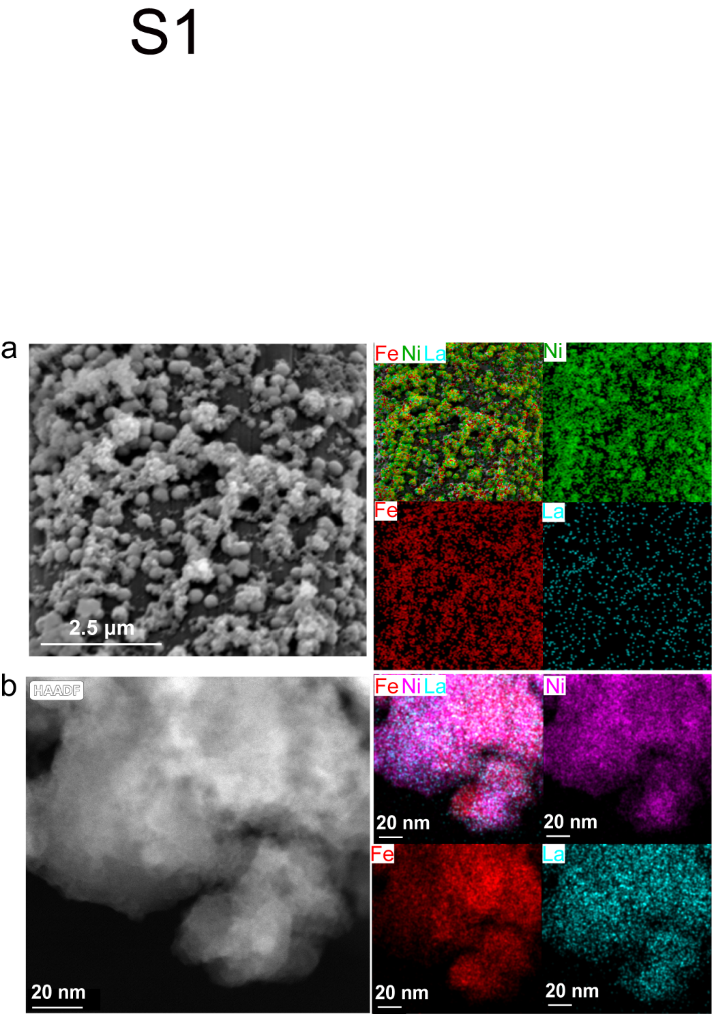


**Fig. S5** EDS with elemental mapping images of NiFeLa


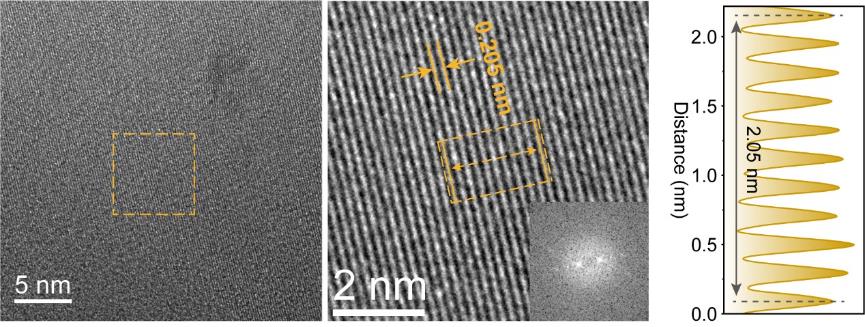


**Fig. S6** TEM image of NiFeMo alloy


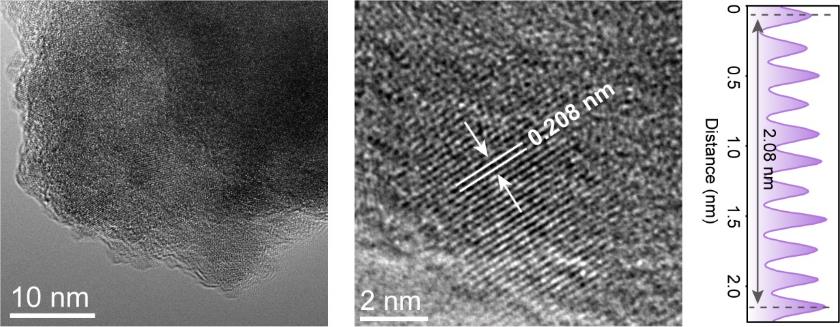


**Fig. S7** TEM image of NiFeMo alloy


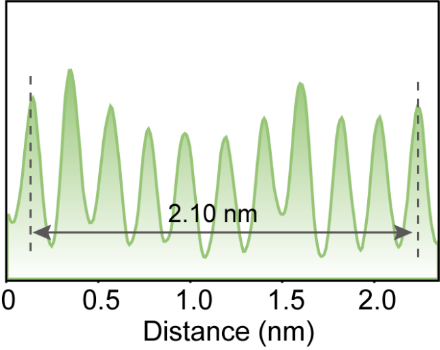


**Fig. S8** The measured atomic distance of NiFeLa labelled in Fig.1f in the manuscript with order region


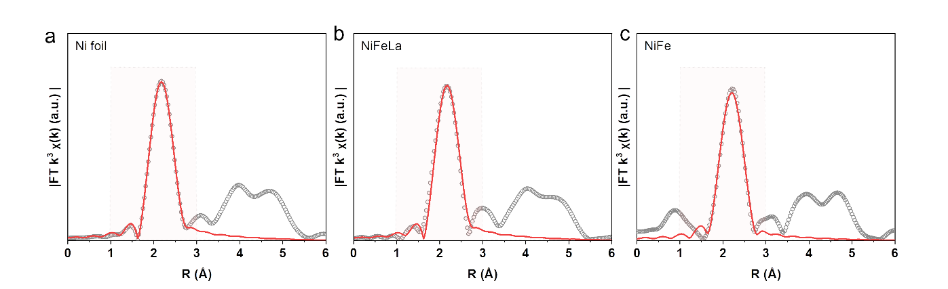


**Fig. S9** The Fourier transform phase corrected using the first shell by Ni K-edge EXAFS data for (**a**) Ni foil; (**b**) NiFeLa; (**c**) NiFe


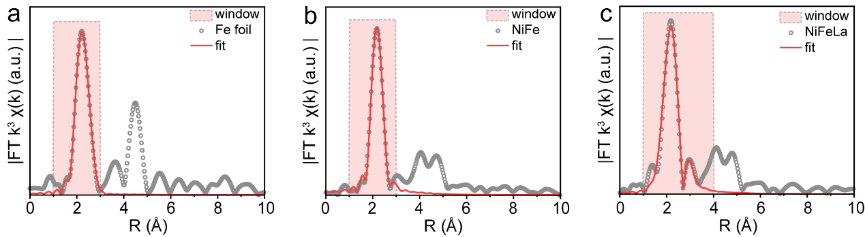


**Fig. S10** The Fourier transform phase corrected using the first shell by Fe K-edge EXAFS data for (**a**) Fe foil; (**b**) NiFe; (**c**) NiFeLa


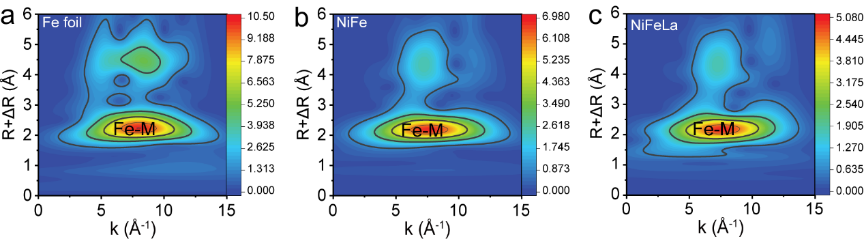


**Fig. S11** The Fourier transform phase corrected using the first shell by Fe K-edge EXAFS data for (**a**) Ni foil; (**b**) NiFe; (**c**) NiFeLa


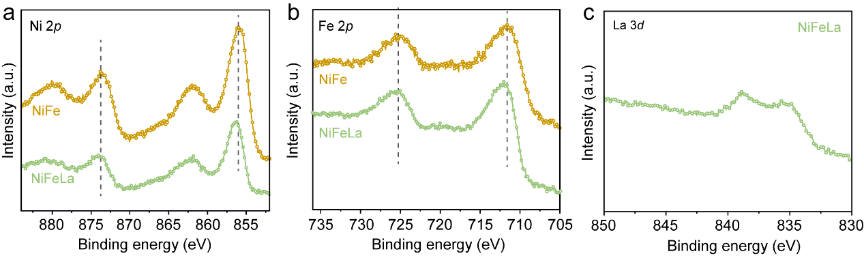


**Fig. S12** (**a**) Ni 2*p* XPS spectra of NiFe and NiFeLa. (**b**) Fe 2*p* XPS spectra of NiFe and NiFeLa. (**c**) La 3*d* XPS spectra of NiFeLa


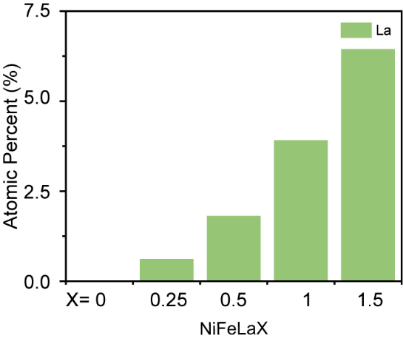


**Fig. S13** The content of La in NiFeLaX (X=0.25, 0.5, 1, 1.5) was determined by ICP-OES


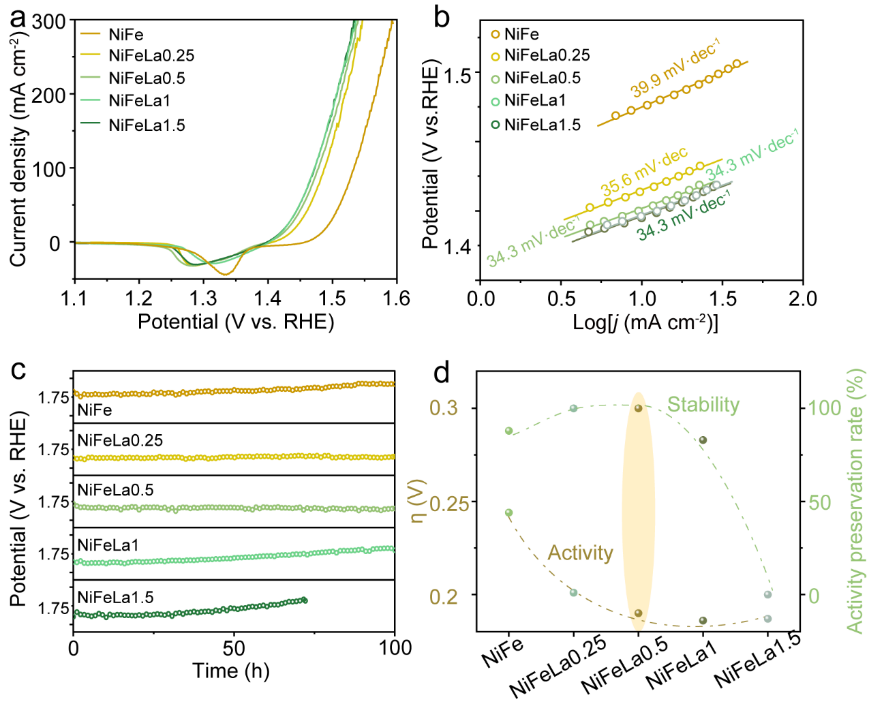


**Fig. S14** (**a**) LSV curves tested in 1 M KOH solution at oxygen-saturated atmosphere. (**b**) Corresponding Tafel plots according to the LSV curves. (**c**) Chronopotentiometric curves at a current density of 1A cm^-2^. (**d**) Comparison of the corresponding activity preservation rate after 100 h and overpotential at the current density of 10 mA cm^-2^


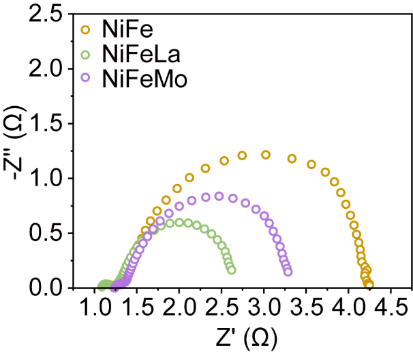


**Fig. S15** Electrochemical impedance spectroscopy of the NiFe, NiFeLa, and NiFeMo


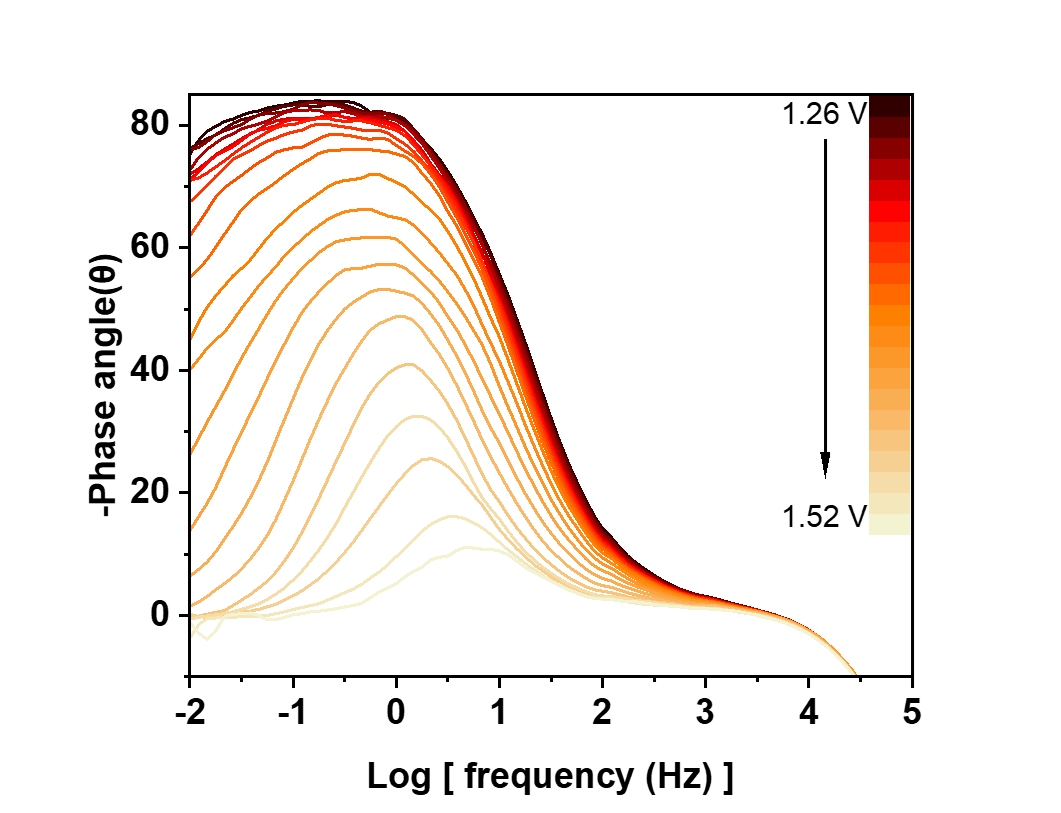


**Fig. S16** Bode phase plots of NiFe


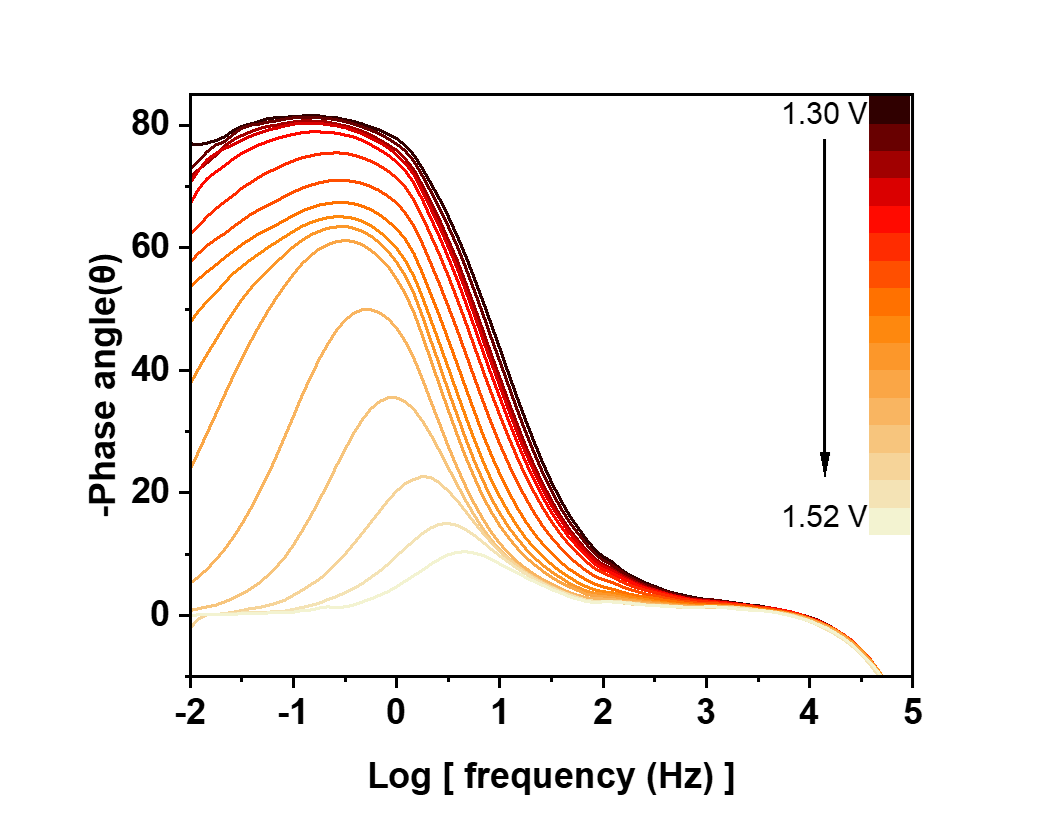


**Fig. S17** Bode phase plots of NiFeMo


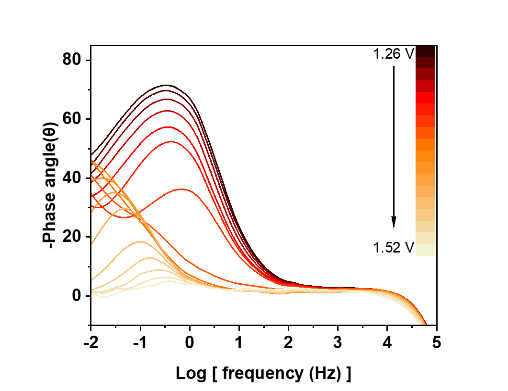


**Fig. S18** Bode phase plots of NiFeLa


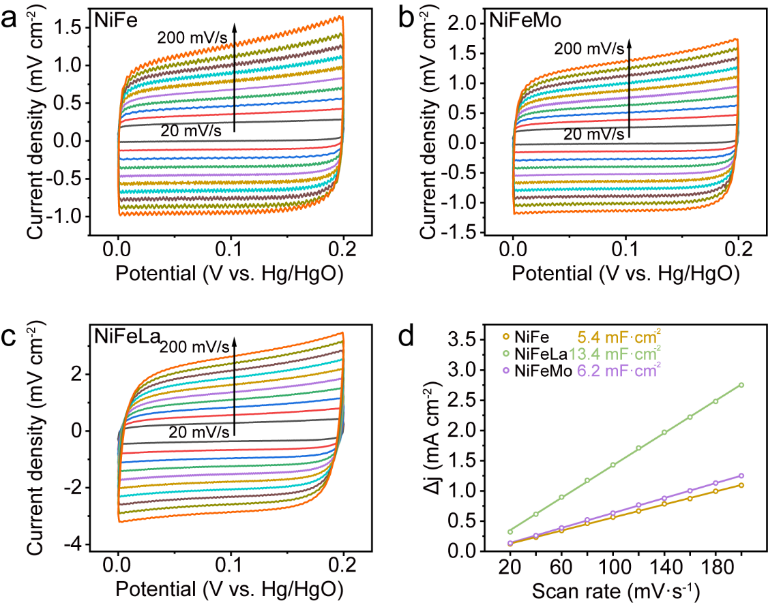


**Fig. S19** CV curves of (**a**) NiFe, (**b**) NiFeMo, (**c**) NiFeLa. (**d**) The C_dl_ values of NiFe, NiFeMo and NiFeLa


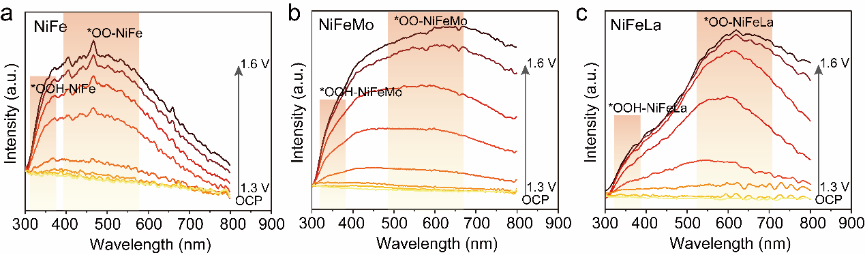


**Fig. S20** Potential-dependent UV–vis spectra of (**a**) NiFe, (**b**) NiFeMo and (**c**) NiFeLa electrodes on indium tin oxide (ITO) at applied potentials from 1.3 to 1.6 V


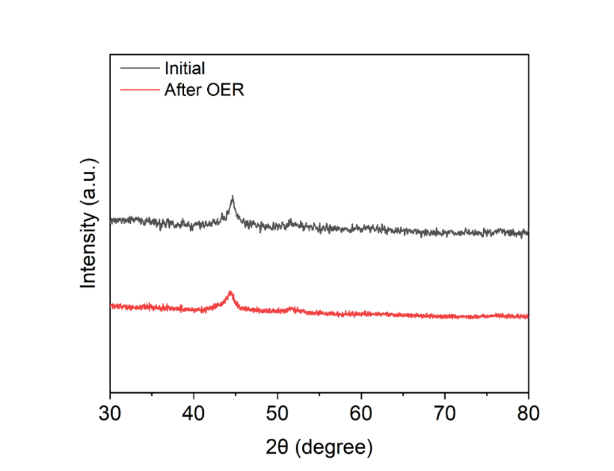


**Fig. S21** The XRD patterns of NiFeLa before and after OER


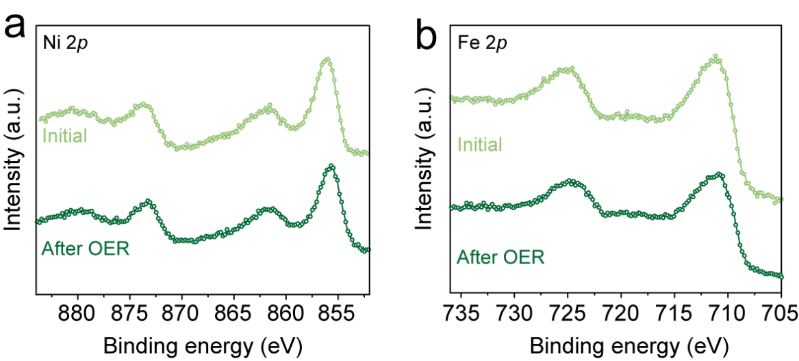


**Fig. S22** (**a**) Ni 2*p* XPS spectra of NiFeLa before and after OER. (**b**) Fe 2*p* XPS spectra of NiFeLa before and after OER


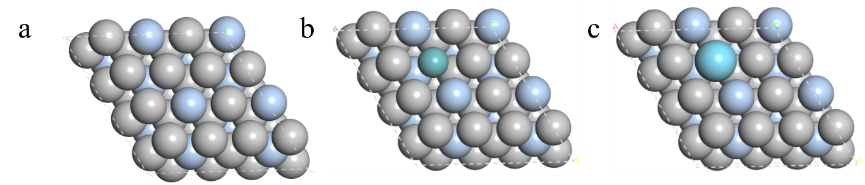


**Fig. S23** The geometric configurations of (**a**) NiFe, (**b**) NiFeMo and (**c**) NiFeLa


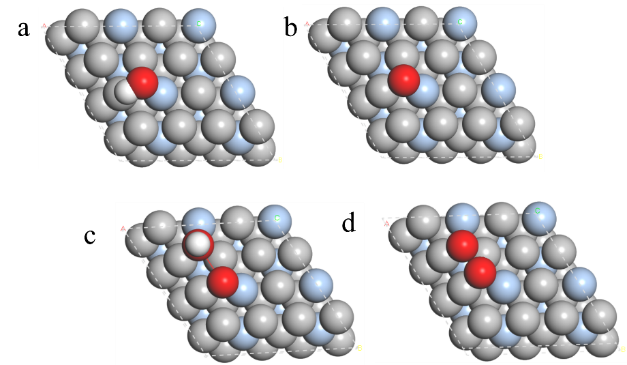


**Fig. S24** The geometric configurations of intermediate adsorption on NiFe


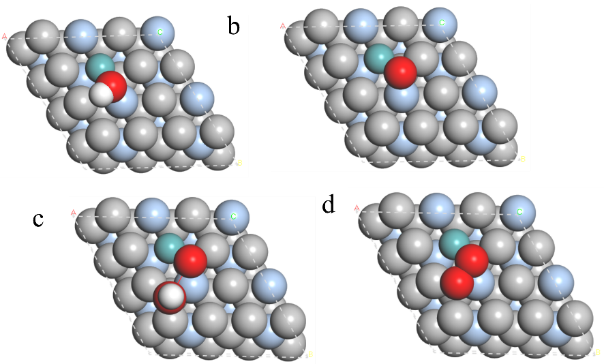


**Fig. S25** The geometric configurations of intermediate adsorption on NiFeMo


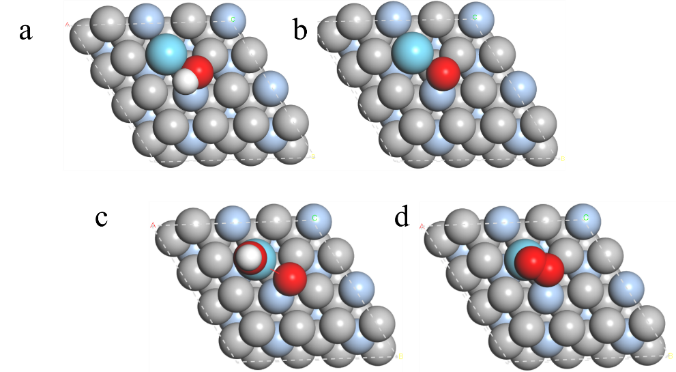


**Fig. S26** The geometric configurations of intermediate adsorption on NiFeLa


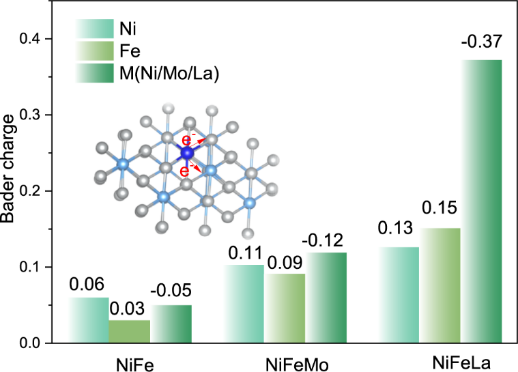


**Fig. S27** The Bader charge of NiFe, NiFeMo and NiFeLa


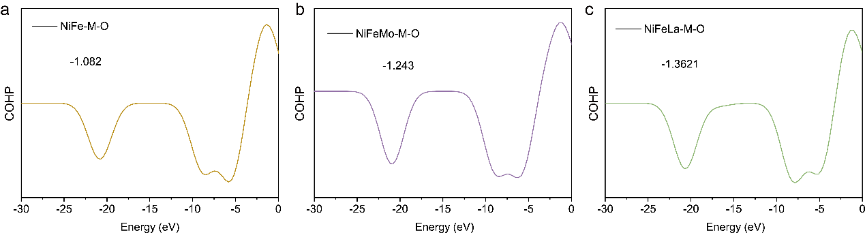


**Fig. S28** The crystal orbital Hamilton population (COHP) analysis of M-O bond in (**a**) *HOO-NiFe, (**b**) *HOO-NiFeMo and (**c**) *HOO-NiFeLa samples

**
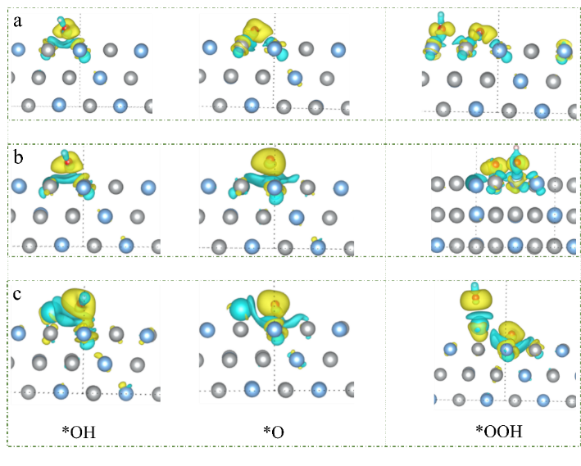
**

**Fig. S29** Electronic charge difference of (**a**) *OH-NiFeLa, *O-NiFeLa and *OOH-NiFeLa; (**b**) *OH-NiFe, *O-NiFe and *OOH-NiFe; (**c**) OH-NiFeMo, *O-NiFeMo and *OOH-NiFeMo


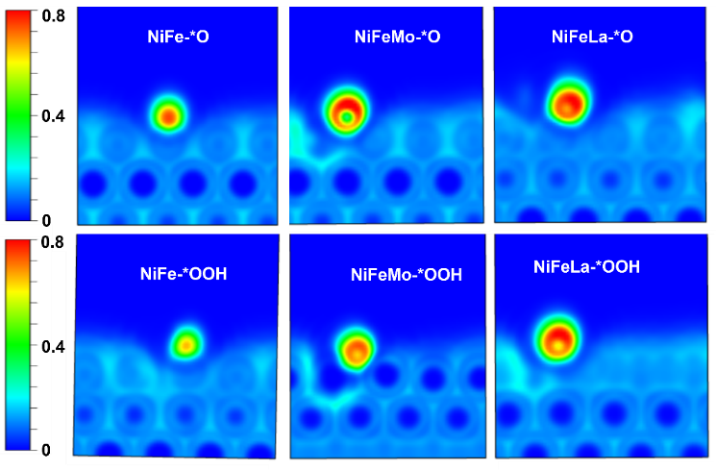


**Fig. S30** Electron localization function (ELF) of *O-NiFe, *O-NiFeMo and *O-NiFeLa; as well as *OOH-NiFe, *OOH-NiFeMo and *OOH-NiFeLa


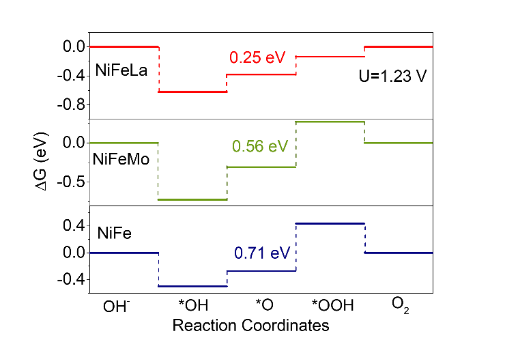


**Fig. S31** Free energy diagram of OER for NiFe, NiFeMo and NiFeLa at U = 1.23V


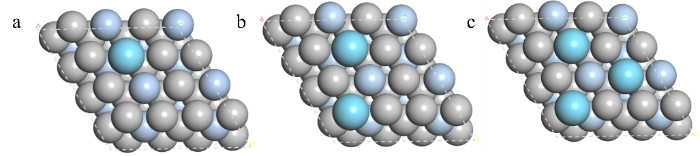


**Fig. S32** The geometric configuration of (**a**) NiFeLa-1 (**b**) NiFeLa-2 and (**c**) NiFeLa-3


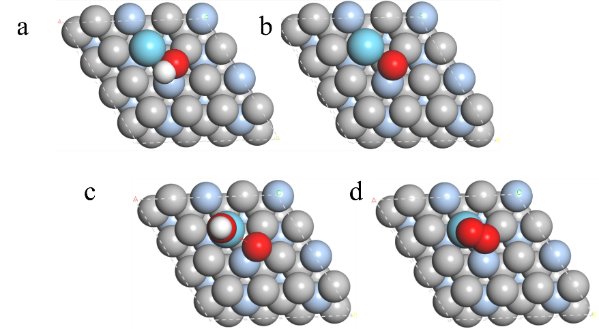


**Fig. S33** The geometric configurations of intermediate adsorption on NiFeLa-1


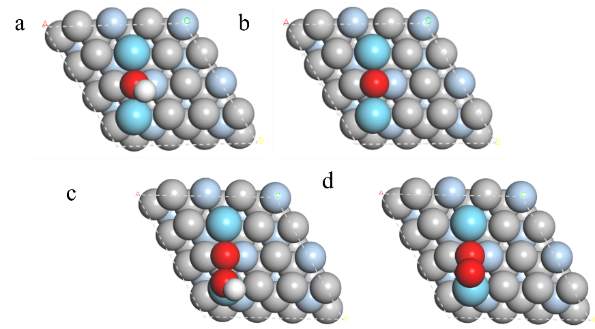


**Fig. S34** The geometric configurations of intermediate adsorption on NiFeLa-2


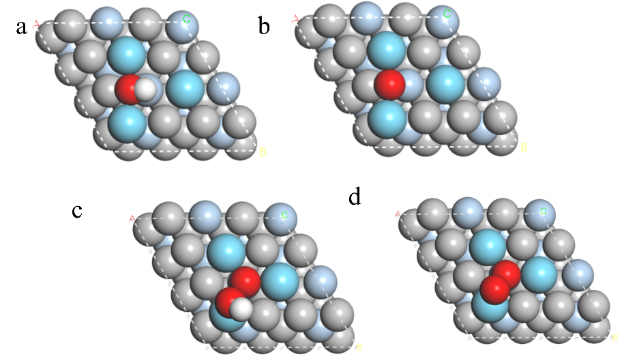


**Fig. S35** The geometric configurations of intermediate adsorption on NiFeLa-3


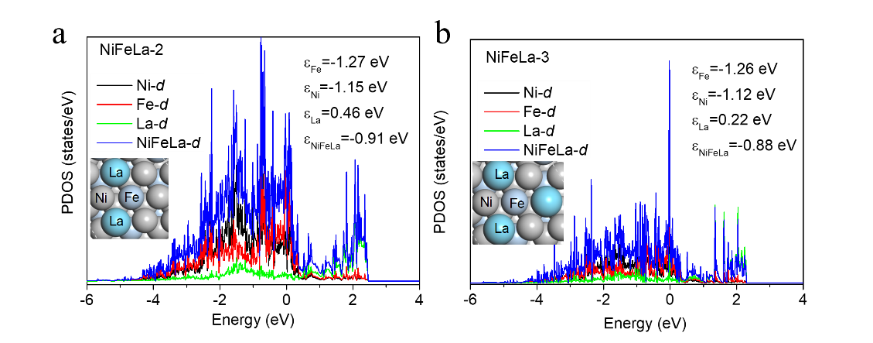


**Fig. S36** The partial density of states (PDOS) of (**a**) NiFeLa-2, (**b**) NiFeLa-3


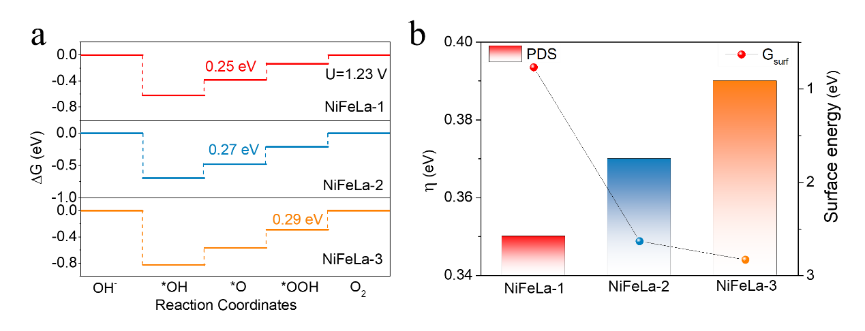


**Fig. S37** (**a**) Free energy diagram of OER for NiFeLa with different La ratio at U = 1.23V. (**b**) The ΔG_PDS_ and ΔG_surf_ analysis in NiFeLa with different La ratio


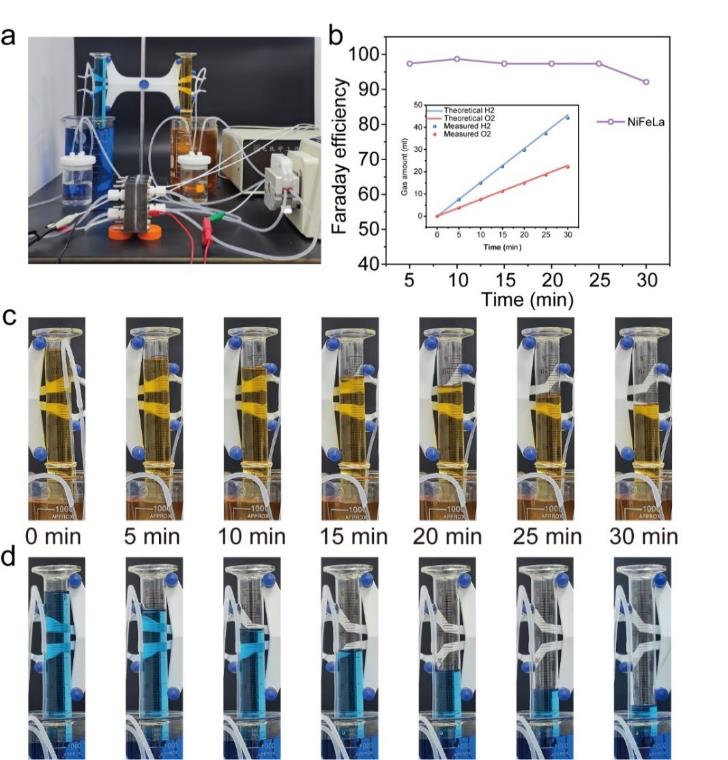


**Fig. S38** (**a**) Photographs of AEM water electrolyzer. (**b**) Faradaic efficiency of NiFeLa. Corresponding levels of oxygen (**c**) and hydrogen (**d**) gas generated at 0, 5, 10, 15, 20, 25 and 30 min

**
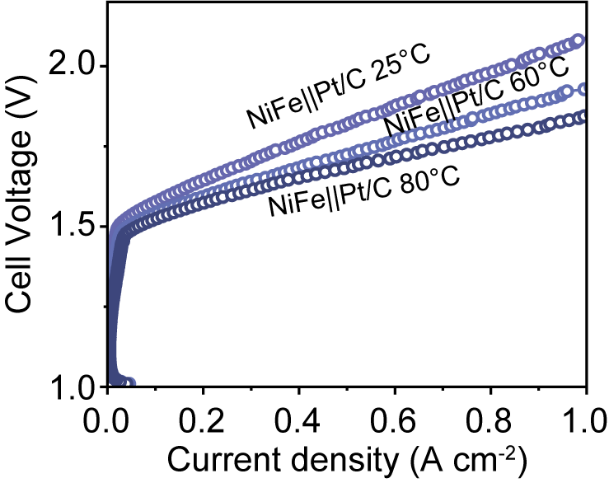
**

**Fig. S39** LSV curves of AEM water electrolyzer for NiFe//Pt/C at 25, 60 and 80 °C

**Table S1** Summary of the fitting parameters of Ni K-edge EXFAS curves for the standard Ni foil, NiFe and NiFeLa alloy. (Ѕ_0_^2^=0.71)

|  | **shell** | **CN** | **R(Å)** | **σ^2^** | **ΔE_0_** | **R factor** |
| --- | --- | --- | --- | --- | --- | --- |
| **Ni foil** | Ni-Ni | 12 | 2.49 | 0.00597 | -7.34±0.57 | 0.0026 |
| **NiFe** | Ni-Ni | 9.37±1.41 | 2.50 | 0.00833 | 10.08±1.50 | 0.0224 |
| **NiFeLa** | Ni-Ni | 7.00±1.37 | 2.48 | 0.00591 | 6.54±2.04 | 0.0319 |

^a^CN: the coordination number; ^b^R: the bond distance; ^c^σ^2^: the Debye-Waller factors; ^d^ΔE_0_: the inner potential correction. R factor: goodness of fitting.

**Table S2** Summary of the fitting parameters of Fe K-edge EXFAS curves for the standard Ni foil, NiFe and NiFeLa alloy. (Ѕ_0_^2^=0.71)

|  | **shell** | **CN** | **R(Å)** | **σ^2^** | **ΔE_0_** | **R factor** |
| --- | --- | --- | --- | --- | --- | --- |
| **Fe foil** | Fe-Fe | 8 | 2.50 | 0.00586 | 5.29±2.43 | 0.0063 |
| **NiFe** | Fe-M | 6.84 | 2.46 | 0.00618 | 5.21±1.23 | 0.0040 |
| **NiFeLa** | Fe-M | 5.37 | 2.41 | 0.00952 | -13.9±9.45 | 0.0289 |

**Table S3** The percentage of metal elements in NiFe based catalyst analyzed

| **Sample** | **Ni** | **Fe** | **La** |
| --- | --- | --- | --- |
| **NiFe** | 68.8% | 31.2% | / |
| **NiFeLa0.25** | 68.2% | 31.2% | 0.6% |
| **NiFeLa0.5** | 66.7% | 31.5% | 1.8% |
| **NiFeLa1** | 65.3% | 30.8% | 3.9% |
| **NiFeLa1.5** | 64.5% | 29.0% | 6.4% |

**Table S4** Comparison of OER performances of NiFeLa with the transiin metal-based electrocatalysts in 1M KOH solution

| Catalyst | Support | Ƞ at 10 mA·cm^-2^ (mV) | Tafel slope (mV dec^-1^) | Refs. |
| --- | --- | --- | --- | --- |
| NiFeLa | CC | 190 | 0.478 | **This work** |
| ZnCo_2_O_4-x_F_x_ | CNTs | 350 | 59.2 | [S3] |
| FeNi_3_ nanocrystal/MG composite | MG | 214 | 32.4 | [S4] |
| DD-Ni-NDA | Carbon paper | 260 | 50 | [S5] |
| LaNi_5_-H-100th | FTO | 291 | 59 | [S6] |
| NiOOH/(LDH/α-FeOOH) | NF | 195 | 35 | [S7] |
| NiCo LDH-TPA | / | 260 | 50 | [S8] |
| CoCuFeMoOOH@Cu | Cu foils | 199 | 48.8 | [S9] |
| CoVFeN@NF | NF | 212 | 34.8 | [S10] |
| NiS_2_–NiOOH | CC | 241 | 51 | [S11] |
| Fe-NiMo-NH_3_/H_2_ | NF | 192 | 28 | [S12] |
| Fe_20_Co_20_Ni_20_Mo_20_Al_20_ | / | 223 | 39.8 | [S13] |
| Hollow Ni/FeMn@CoNiSe nanocages | NF | 184 | 43.4 | [S14] |
| FeOOH/Co(OH)_2_ | Co foam | 265 | 38 | [S15] |
| d-(Fe,Ni)OOH | Fe foam | 213 | 21.7 | [S15] |

**Table S5** Comparison of ECSA-normalized specific activity of NiFe, NiFeMo and NiFeLa

| Catalyst | C_dl_ (mF·cm^-2^) | Current density (mA·cm^-2^_ECSA_) at 1.45 V | Current density (mA·cm^-2^_ECSA_) at 1.5 V |
| --- | --- | --- | --- |
| NiFe | 5.4 | / | 0.234 |
| NiFeMo | 6.2 | 0.011 | 0.284 |
| NiFeLa | 13.4 | 0.128 | 0.478 |

**Table S6** Comparison the current density of AEM water electrolyzer for the NiFeLa0.5||Pt/C cell and the reported AEM water electrolyzer cells at the current density of 1 A cm^-2^

| Catalyst | Temperature (ºC) | Cell voltage (V) | Stability (h) | Refs. |
| --- | --- | --- | --- | --- |
| NiFeLa0.5\|\|Pt/C | 80 | 1.58 | 600 | This work |
| Fe-NiCo_2_S_4_ @NIF\|\|Pt/C | 25 | 1.83 | 100 | [S16] |
| Fe-NiMo-NH_3_/H_2_\|\|NiMo-NH_3_/H_2_ | 80 | 1.57 | 25 | [S12] |
| NiFe_FA_NN\|\|NiFeP_FA_NN | 60 | 1.73 | 200 | [S17] |
| Co, Mo-NiFe LDH \|\| Pt/C | 25 | 1.94 | 130 | [S18] |
| Ni_2_Fe_8_–Ni_3_S_2_/NF \|\|Ni_4_Mo/MoO_2_/NF | 80 | 1.65 | 100 | [S19] |
| Ru@Cu-TM \|\| 5-stacked SSMs AEL | 80 | 1.69 | 300 | [S20] |
| (Fe,Ni)OOH\|\|NiMoN | 70 | ~1.85 | 96 | [S15] |
| NiFe LDH \|\| Pt/C | 80 | 1.59 | 6 | [S21] |
| Ni\|\|Ni_0.8_Fe_0.2_-AHNA | 25 | 1.76 | 100 | [S22] |
| Ir@Zr–CoP\|\| Ir@Zr–CoP | 60 | 1.88 | 150 | [S23] |
| NiFe_2_O_4_\|\| Pt/C | 60 | 1.78 | 72 | [S24] |

**Supplementary References**

1. D. Liu, Y. Yan, H. Li, D. Liu, Y. Yang et al., A template editing strategy to create interlayer-confined active species for efficient and durable oxygen evolution reaction. Adv. Mater. **35**, 2203420 (2023). <https://doi.org/10.1002/adma.202203420>
2. G. Kresse, J. Furthmüller Efficiency of ab-initio total energy calculations for metals and semiconductors using a plane-wave basis set. Comput. Mater. Sci. **6**, 15–50 (1996). <https://doi.org/10.1016/0927-0256(96)00008-0>
3. K. Xiao, Y. Wang, P. Wu, L. Hou, Z. Q. Liu, Activating lattice oxygen in spinel ZnCo(2)O(4) through filling oxygen vacancies with fluorine for electrocatalytic oxygen evolution. Angew. Chem. Int. Ed. **62**(24), e202301408 (2023). <https://doi.org/10.1002/anie.202301408>
4. R. Li, R. Wu, Z. Li, J. Wang, X. Liu et al., Boosting oxygen-evolving activity via atom-stepped interfaces architected with kinetic frustration. Adv. Mater. **35**(50), e2206890 (2023). <https://doi.org/10.1002/adma.202206890>
5. Y. Liu, X. Li, S. Zhang, Z. Wang, Q. Wang et al., Molecular engineering of metal-organic frameworks as efficient electrochemical catalysts for water oxidation. Adv. Mater. **35**(22), e2300945 (2023). <https://doi.org/10.1002/adma.202300945>
6. Z. Chen, H. Yang, S. Mebs, H. Dau, M. Driess et al., Reviving oxygen evolution electrocatalysis of bulk la-ni intermetallics via gaseous hydrogen engineering. Adv. Mater. **35**(11), e2208337 (2023). <https://doi.org/10.1002/adma.202208337>
7. M. Cai, Q. Zhu, X. Wang, Z. Shao, L. Yao et al., Formation and stabilization of niooh by introducing alpha-feooh in ldh: Composite electrocatalyst for oxygen evolution and urea oxidation reactions. Adv. Mater. **35**(7), e2209338 (2023). <https://doi.org/10.1002/adma.202209338>
8. W. Liu, D. Zheng, T. Deng, Q. Chen, C. Zhu et al., Boosting electrocatalytic activity of 3d-block metal (hydro)oxides by ligand-induced conversion. Angew. Chem. Int. Ed. **60**, 10614–10619 (2021). <https://doi.org/10.1002/anie.202100371>
9. L. Zhang, W. Cai, N. Bao, Top-level design strategy to construct an advanced high-entropy co-Cu-Fe-Mo (oxy)hydroxide electrocatalyst for the oxygen evolution reaction. Adv. Mater. **33**, e2100745 (2021). <https://doi.org/10.1002/adma.202100745>
10. D. Liu, H. Ai, J. Li, M. Fang, M. Chen et al., Surface reconstruction and phase transition on vanadium–cobalt–iron trimetal nitrides to form active oxyhydroxide for enhanced electrocatalytic water oxidation. Adv. Energy Mater. **10**(45), 2002464 (2020). <https://doi.org/0.1002/aenm.202002464>
11. H. Zhong, X. Wang, G. Sun, Y. Tang, S. Tan et al., Optimization of oxygen evolution activity by tuning e*g band broadening in nickel oxyhydroxide. Energy Environ Sci. **16**(2), 641-652 (2023). <https://doi.org/10.1039/d2ee03413a>
12. P. Chen, X. Hu, High-efficiency anion exchange membrane water electrolysis employing non-noble metal catalysts. Adv. Energy Mater. **10**, 2002285 (2020). <https://doi.org/10.1002/aenm.202002285>
13. Y.F. Cui, S.D. Jiang, Q. Fu, R. Wang, P. Xu et al., Cost‐effective high entropy core–shell fiber for stable oxygen evolution reaction at 2 a cm−2. Adv. Funct. Mater. **33**(50), 2306889 (2023). <https://doi.org/10.1002/adfm.202306889>
14. S. Wang, W. Huo, H. Feng, Z. Xie, J. K. Shang et al., Enhancing oxygen evolution reaction performance in prussian blue analogues: Triple-play of metal exsolution, hollow interiors, and anionic regulation. Adv. Mater. **35**(45), e2304494 (2023). <https://doi.org/10.1002/adma.202304494>
15. L. Wu, M. Ning, X. Xing, Y. Wang, F. Zhang et al., Boosting oxygen evolution reaction of (Fe, Ni)OOH via defect engineering for anion exchange membrane water electrolysis under industrial conditions. Adv. Mater. **35**, e2306097 (2023). <https://doi.org/10.1002/adma.202306097>
16. F.-L. Wang, Y.-W. Dong, C.-J. Yu, B. Dong, X.-Y. Zhang et al., Trojan strategy assisted phase-pure Fe-NiCo_2_S_4_ for industrial anion-exchange membrane water electrolyzer. Appl. Catal. B **331**, 122660 (2023). <https://doi.org/10.1016/j.apcatb.2023.122660>
17. Z. Wei, M. Guo, Q. Zhang, Scalable electrodeposition of nife-based electrocatalysts with self-evolving multi-vacancies for high-performance industrial water electrolysis. Appl. Catal. B **322**, 122101 (2023). <https://doi.org/10.1016/j.apcatb.2022.122101>
18. Y. Zhao, Q. Wen, D. Huang, C. Jiao, Y. Liu et al., Operando reconstruction toward dual‐cation‐defects co‐containing nife oxyhydroxide for ultralow energy consumption industrial water splitting electrolyzer. Adv. Energy Mater. **13**(10), 2203595 (2023). <https://doi.org/10.1002/aenm.202203595>
19. G. Ding, H. Lee, Z. Li, J. Du, L. Wang et al., Highly efficient and durable anion exchange membrane water electrolyzer enabled by a Fe–Ni_3_S_2_ anode catalyst. Adv. Energy Sustain. Res. **4**(1), 2200130 (2022). <https://doi.org/10.1002/aesr.202200130>
20. Y. Zuo, S. Bellani, M. Ferri, G. Saleh, D. V. Shinde et al., High-performance alkaline water electrolyzers based on ru-perturbed cu nanoplatelets cathode. Nat. Commun. **14**(1), 4680 (2023). <https://doi.org/10.1038/s41467-023-40319-5>
21. H. Koshikawa, H. Murase, T. Hayashi, K. Nakajima, H. Mashiko et al., Single nanometer-sized nife-layered double hydroxides as anode catalyst in anion exchange membrane water electrolysis cell with energy conversion efficiency of 74.7% at 1.0 a cm^–2^. ACS Catal. **10**(3), 1886-1893 (2020). <https://doi.org/10.1021/acscatal.9b04505>
22. C. Liang, P. Zou, A. Nairan, Y. Zhang, J. Liu et al., Exceptional performance of hierarchical Ni–Fe oxyhydroxide@NiFe alloy nanowire array electrocatalysts for large current density water splitting. Energy Environ. Sci. **13**(1), 86-95 (2020). <https://doi.org/10.1039/C9EE02388G>
23. Q.P. Ngo, T.T. Nguyen, Q.T.T. Le, J.H. Lee, N.H. Kim, Unveiling the synergistic effect of atomic iridium modulated zirconium-doped pure phase cobalt phosphide for robust anion-exchange membrane water electrolyzer. Adv. Energy Mater. **13**, 2301841 (2023). <https://doi.org/10.1002/aenm.202301841>
24. A. Martinez-Lazaro, A. Caprì, I. Gatto, J. Ledesma-García, N. Rey-Raap et al., NiFe_2_O_4_ hierarchical nanoparticles as electrocatalyst for anion exchange membrane water electrolysis. J. Power Sources **556,** 232417 (2023). <https://doi.org/10.1016/j.jpowsour.2022.232417>
